# Salinity matters the most: How environmental factors shape the diversity and structure of cyanobacterial mat communities in high altitude arid ecosystems

**DOI:** 10.3389/fmicb.2023.1108694

**Published:** 2023-04-13

**Authors:** Małgorzata Sandzewicz, Nataliia Khomutovska, Łukasz Łach, Jan Kwiatowski, Toirbek Niyatbekov, Małgorzata Suska-Malawska, Iwona Jasser

**Affiliations:** ^1^Institute of Environmental Biology, Faculty of Biology, Biological and Chemical Research Centre, University of Warsaw, Warsaw, Poland; ^2^Institute of Botany, Plant Physiology and Genetics, Academy Science Republic of Tajikistan, Dushanbe, Tajikistan

**Keywords:** benthic cyanobacteria, microbial mats, cold mountain desert, Eastern Pamir, 16S rRNA gene, V3-V4 hypervariable region

## Abstract

**Introduction:**

Microbial mats are complex communities of benthic microorganisms that occur at the soil-water interphase in lakes’ shores, streams, and ponds. In the cold, mountainous desert of Eastern Pamir (Tajikistan), where scarce water bodies are influenced by extreme environmental conditions, photosynthetic cyanobacteria form diverse mats. The mats are characterized by different morphology and thickness. Their habitats exhibit a wide range of conditions; from oligosaline to hypersaline, oligotrophic to hypertrophic, and from cold ponds to hot springs. The aim of the present study was to reveal the taxonomic composition and structure of these mats and to examine which environmental factors influence them.

**Methods:**

Fifty-one mats were collected from small water bodies around Bulunkul, Karakul, and Rangkul Lakes in 2015 and 2017. The physical and chemical properties of the water were measured in situ, while the concentration of nutrients was analyzed ex-situ. To reveal the taxonomic composition of the mats, the hypervariable V3-V4 region of the 16S rRNA gene was examined using NGS technology.

**Results:**

The results of bioinformatic analyses were compared with microscopic observations. They showed that Cyanobacteria was the dominant phylum, constituting on average 35% of bacterial ASVs, followed by Proteobacteria (28%), Bacteroidota (11%), and Firmicutes (9%). Synechococcales, Oscillatoriales, and Nostocales orders prevailed in Oxyphotobacteria, with a low contribution of Chroococcales, Gloeobacterales, and Chroococcidiopsidales. Occasionally the non-photosynthetic Vampirivibrionia (Melainabacteria) and Sericytochromatia from sister clades to Oxyphotobacteria were noted in the samples. Moreover, there was a high percentage of unidentified cyanobacterial sequences, as well as the recently described *Hillbrichtia pamiria* gen. et sp. nov., present in one of the samples. Salinity, followed by Na and K concentrations, correlated positively with the composition and structure of Oxyphotobacteria on different taxonomic levels and the abundance of all bacterial ASVs.

**Discussion:**

The study suggests that the investigated communities possibly host more novel and endemic species. Among the environmental factors, salinity influenced the Oxyphotobacteria communities the most. Overall, the microenvironmental factors, i.e. the conditions in each of the reservoirs seemed to have a larger impact on the diversity of microbial mats in Pamir than the “subregional” factors, related to altitude, mean annual air temperature and distance between these subregions.

## 1. Introduction

Deserts have been colloquially associated with barren lands, worn out by the scorching sun and hostile to their inhabitants. However, just because some conditions are less favorable for humans, does not mean that other organisms cannot thrive within them. Around 40% of the Earth’s land surface is covered by arid, semi-arid and dry sub-humid environments (Fraser et al., 2011), from arctic regions of the poles to the intertropical drylands. Each ecosystem is unique in terms of its topography, extreme climatic conditions, and abundance of divergent microbial life.

Among the organisms that perfected their pioneering abilities in desert environments are cyanobacteria. They are common in almost all environments but their role in desert microbial consortia is especially significant. They can perform oxygenic photosynthesis, in high light and high UV intensity as well as low light conditions (Castenholz and Garcia-Pichel, 2012; Stal, 2012). In addition, many cyanobacteria can fix atmospheric nitrogen (N_2_). They can colonize the upper layer of soil, surface and rocks’ matrix, sediments, and water reservoirs. As primary producers, cyanobacteria help diversify ecosystems and provide necessary conditions for the development of more complex communities (Hagemann et al., 2015). These communities can take the form of microbial mats - multilayered associations made of archaea, bacteria, eukaryotic algae, and other eukaryotes, for example diatoms, lichens, and mosses (Stal, 2012; Prieto-Barajas et al., 2018). Microbial mats grow on the borderline of terrestrial and aquatic environments, such as sediments of shallow pools, streams, and lake shores. Cyanobacteria are components of these communities and cannot be overestimated, as apart from carbon and nitrogen fixation they produce exopolysaccharides (EPS) that are essential for the functioning of mats, binding mat layers, providing structure and stability, as well as a medium for other bacteria (Stal, 2012; Bolhuis et al., 2014). Microbial mats are common in arid and semi-arid environments in which lakes with various salinity and turbidity levels and surrounding wetlands occur and are very important for the functioning of these fragile ecosystems (Vincent et al., 1993; Stal, 2012). Microbial mats also are the first terrestrial ecosystems in early life on earth which paved the way for the evolution of such land ecosystems to complex, plant-dominated ones (Beraldi-Campesi, 2013).

A large variety of different types of microbial mats can be found in Eastern Pamir (Khomutovska et al., 2020). It is a cold, arid and highly elevated plateau with low vegetation (Breckle and Wucherer, 2006; Mętrak et al., 2015). In previous studies, three subregions in Eastern Pamir were distinguished for the purpose of comparing cyanobacterial and bacterial diversity in endoliths and biological soil crusts. They were areas around lakes Bulunkul, Karakul, and Rangkul (Khomutovska et al., 2021a). This division was based on the geographical distance between the lakes and the presence of high mountain ranges between sampled subregions, causing their partial isolation as well as hydrological and geological differences as they formed or belonged to different watersheds. From available scarce meteorological data, it seems that Bulunkul area is a cold subregion with mean annual temperatures of −4.3°C and mean precipitation of 59 mm in the years between 2009–2014 (Mętrak et al., 2017). The lowest winter temperature noted in the area of Lake Bulunkul was −63°C and it was also the lowest temperature registered in the Eastern Pamir (Mętrak et al., 2015). Rangkul area seems to be the warmest subregion, with mean annual temperatures of −0.7°C and 74 mm of mean annual precipitation in the years 1950–2005 (Mętrak et al., 2017). The mean temperatures and precipitation recorded for the Karakul area were, respectively, 3.9°C and 82 mm (Aichner et al., 2015) and −3.8°C and 80 mm (Kabala et al., 2021).

The importance of studies of dryland ecosystems is widely discussed since these are the areas where the results of environmental changes are rapidly progressing (Fraser et al., 2011). In the Pamir Mountains, the change of vegetation cover resulting from global climate change have also been recorded (Mętrak et al., 2015). Together with the problem of harmful anthropogenic impact, these factors can lead to a decline of the biodiversity of Pamirian ecosystems (Breckle and Wucherer, 2006). With the changes being so unpredictable it is important to learn as much as possible about the taxonomic composition of microorganisms in cyanobacterial mats and their relation to the environment. Previously, the studies on the diversity of cyanobacteria in Eastern Pamir mostly concerned planktic species and they were solely based on morphological identification (Niyatbekov, 2006; Barinova et al., 2016; Barinova and Niyatbekov, 2018). Other works focused on benthic biofilms and cyanobacterial inocula in small water bodies and lakes (Jasser et al., 2019), further on cyanotoxin production potential in microbial mats (Khomutovska et al., 2020) and on endolithic and soil communities (Khomutovska et al., 2017, 2021a,b). To our knowledge, the work by Khomutovska et al. (2020), mentioned above, is to this day the only research about cyanobacterial mats in Pamir Mountains, Tajikistan. Having discovered very high variability and biomass of microbial mats in various water bodies in Eastern Pamir Mountains and an indication of new species and new secondary metabolites we decided to study closer the diversity of cyanobacteria and other bacteria forming these mats as well as environmental factors influencing this diversity.

Thus, the objectives of this study are: (1) to reveal the cyanobacterial diversity and taxonomic structure of different types of microbial mats of Eastern Pamir Mountains; (2) and to search for geographic (distance and subregional division) and microenvironmental factors (physical and chemical parameters of studied water bodies) affecting the diversity and taxonomic structure of microbial communities. Based on previous studies concerning the composition and structure of cyanobacteria inocula in the upper layer of sediments in small water bodies in the Pamir Mountains (Jasser et al., 2019) we put forward two hypotheses which we wanted to verify in this work. The first one states that the microenvironmental parameters of different reservoirs related to the concentration of nutrients in water, its pH, salinity, and temperature of the water will have a larger impact on microbial structure than the geographic distance between studied areas. Salinity is an environmental chemical parameter that is considered extremely important for microorganisms and especially cyanobacteria and heterotrophic bacteria as it was shown in many publications concerning planktic cyanobacteria (Silveira and Odebrecht, 2019) and soil microorganisms (Kirkwood et al., 2008; Wang et al., 2011) as well as benthic cyanobacteria (Jungblut et al., 2005; Bolhuis et al., 2014; Oren, 2015). The authors of the cited publication demonstrated enhanced germination of akinetes and toxin production by *Nodularia spumigena* in more saline environments regardless of nutrient concentration and temperature. Also, our previous work on benthic cyanobacteria inocula in small water bodies in Eastern Pamir demonstrated that salinity, followed by temperature, were the most important factors correlating with the composition of studied communities (Jasser et al., 2019). Thus, based on this knowledge as well as on the results of other authors we put forward the second hypothesis. This hypothesis states that water salinity is the factor that affects the microbial structure of cyanobacterial mats the most.

## 2. Materials and methods

### 2.1. Environmental data collection, sample collection, and *in situ* preparation

**Figure 1:**
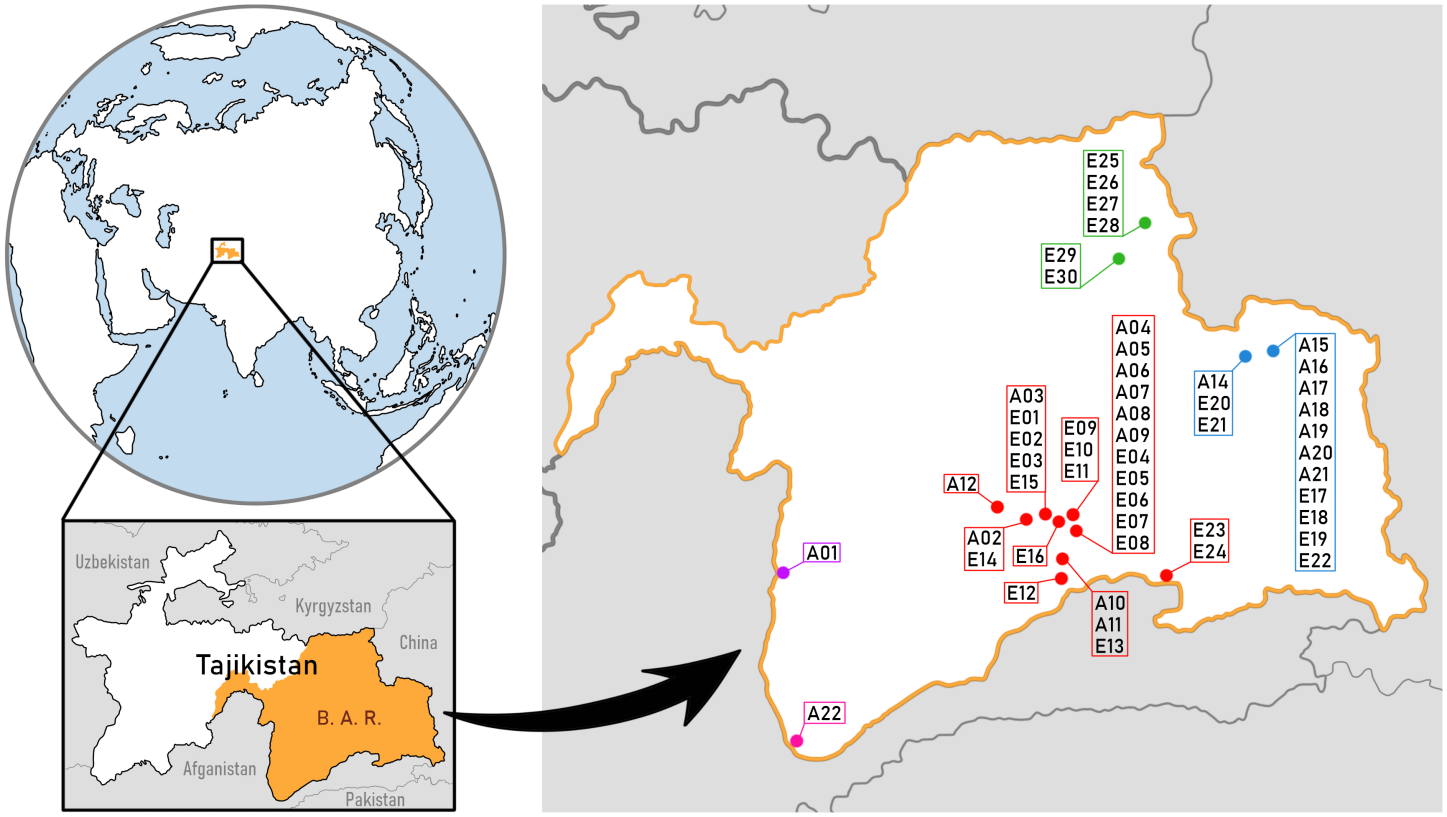
Map of Badakhshan Autonomous Region (B. A. R. on map) in Tajikistan, Central Asia. Sample sites, along with the sample names, are marked by the subregion: Bulunkul in red color, Rangkul in blue, Karakul in green. Samples that were collected outside of the three subregions are the ones from Khorog (marked in violet) and Ishkashim (marked in pink). The map was based on public domain pictures from WikiMedia (https://commons.wikimedia.org).

A total of 51 mat samples were collected from various reservoirs located in the Badakhshan Autonomous Region in Tajikistan. 21 of them (A01-A22) were collected in July 2015 and 30 (E01-E30) in July 2017 (Figure 1). 49 mats were obtained from the three distinguished subregions: (1) Bulunkul - the largest area, with 29 samples collected, containing surroundings of lakes Bulunkul, Yashilkul, Sassykkul, Chukurkul, Khargush, and Zorkul as well as reservoirs near village Alichur; (2) Karakul – an area containing reservoirs surrounding Lake Karakul, with 6 samples collected; (3) Rangkul – an area containing reservoirs surrounding Lakes Rangkul and Shorkul, with 14 samples collected. The two remaining mat samples were gathered from urban areas outside these three subregions, one in Khorog and the second one in Ishkashim and were not included in statistical and bioinformatic analyses of the subregions. Similar mat types were collected randomly from studied subregions, whenever observed. Water from reservoirs from which the samples were obtained, was analyzed *in situ* in terms of temperature, pH, and electrical conductivity, with the use of a HachHQ40d portable multimeter. Samples of water from studied reservoirs were collected into sterile sample tubes and stored in a portable freezer powered by a car battery. They were kept frozen throughout the shipment and later in the laboratory until the *ex-situ* analyses. Geographical location (longitude, latitude) as well as altitude of sample sites was measured using a GPS signal receiver [GarmineTrex 10 (010–00970-00)] and multifunctional mobile navigation app LocusMap. Microbial mat samples were divided into two parts. The first part was dried in the open air, in shadow, screened from wind (but covered with dense mesh) and stored in Petri dishes and the second part was preserved in DESS, a DMSO/NaCl/EDTA mixture which proved to fix well animal tissues (Michaud and Foran, 2011) and bacteria (Gray et al., 2013) allowing for their DNA analysis. Our previous study confirmed that DESS fixation allowed not only for DNA isolation from microbial mats but also for microscopic analysis of cyanobacteria forming those mats. Half of the dried samples were further divided for those which were stored in cool dark conditions for ongoing laboratory analyses and those kept in the freezer.

### 2.2. Laboratory analyses

Sample preparation *ex situ* included reviving the dried samples for cyanobacteria isolation and cultivation. Small parts of dried mats were placed on a solid medium WC (Guillard and Lorenzen, 1972) and BG11 (Rippka et al., 1979) inside a Petri dish. Different cyanobacteria colonies were then isolated and cultivated on separate Petri dishes as well as in liquid media. The cultures were stored in a plant growth chamber at a temperature of 15°C with a 12/12 h day/night period. Microscopic identification of cyanobacteria was performed with a light and epifluorescence microscope [Nikon ECLIPSE Ni-U 930941 (Tokyo, Japan)] using keys by Komárek and Anagnostidis (1998, 2005) and Komárek (2013). The material used in the morphological analysis was both the isolated cyanobacteria strains as well as environmental samples preserved in DESS solution.

Nutrient concentration analysis was performed by the Laboratory of Biogeochemistry and Environmental Conservation of the University of Warsaw. N/C 3100 analyzer (Analytik Jena) was used to analyze total organic carbon (C organic) and total nitrogen (N total). For the analysis of the PO_4_^3−^, NH_4_^+^ and NO_2_^−^ + NO_3_^−^ concentration, the continuous flow analyzer San++ Skalar was used. The cations Na^+^, K^+^ Ca^+2^, and Mg^+2^ were analyzed by a flame atomic absorption spectroscopy (FAAS) ContrAA700 (Analytik Jena). The total phosphorus (P total) concentration was detected with the use of a Hach analysis kit (Sulwiński et al., 2020).

### 2.3. DNA extraction and sequencing

Both dried material, as well as material preserved in DESS solution, was homogenized and prepared for DNA extraction. The extraction was performed using solutions and protocols of the two following kits: E.Z.N.A.^®^ Soil DNA Kit (Omega Bio-tek, Norcross, GA, United States) and Soil DNA Purification Kit (GeneMATRIX, EURx Ltd., Gdańsk, Poland). The process was repeated multiple times, using each extraction kit, until a satisfactory amount and quality of DNA was obtained (Khomutovska et al., 2020, 2021a). The amplification of the V3-V4 region of 16S rRNA was performed with the use of 341f and 785r primers (Klindworth et al., 2012) and HotStarTaq DNA Polymerase.^1^ The reaction mix comprised of 15.1 μL of deionized water, 2.5 μL of PCR buffer, 5 μL of Q-solution, 1 μL of extracted DNA, 0.5 μL of each primer, 0.3 μL of dNTP mix and 0.1 mL of Taq polymerase. The amplification began with 3 min of initial denaturation (95°C) followed by 30 cycles of DNA denaturation (15 s in 95°C), primer attachment (35 s in 53°C) and elongation (1 min in 72°C). The final elongation lasted for 10 min (72°C). The PCR product was sent to the Science and Technology Park “Bionanopark” (Łódź, Poland) for sequencing. It was performed using the Illumina MiSeq platform (2 × 300), with 150,000 targeted sequencing depth. Raw data are available in BioProject database under numbers PRJNA486727 and PRJNA609145.

### 2.4. 16S rRNA gene amplicon analysis and statistical analyses

The bioinformatic and phylogenetic analyses were conducted as described by Khomutovska et al. (2020, 2021a). For processing paired-end reads, QIIME2 environment (ver. 2020.11) was used. Data was denoised and trimmed with the DADA2 method. Taxonomic assignment of the amplicon sequence variants (ASVs) was performed using the SILVA-based classifier (ver. 138) for QIIME2. The downstream analysis was carried out on the rarefied data. The bacterial sequences were further analyzed statistically using R language (ver. 4.0.0) in RStudio (ver. 1.2.5042). Alpha diversity (vegan package), PCA (FactoMineR, factoextra), cluster dendrogram (base R functions hclust() and plot.hclust() from stats package, as well as magrittr package), correlation matrix (corrplot, ggpubr) and numbers of unique and common sequences for each subregion were calculated from this dataset.

To assess the cyanobacterial structure of mats on the order level, the amplicon sequences were verified with Cydrasil v.2 reference alignment and tree (Roush et al., 2016, 2021). The names of the taxa and taxonomic position were verified according to Komárek et al. (2014) and Algaebase (Guiry and Guiry, 2013). This additional verification step was applied to validate the name of taxa obtained after the SILVA-based classifier developed for the QIIME2 environment. That is because the semi-automatic classification was not always correct, which was especially common at the higher taxonomic ranks.

For the heat map (base R function heatmap() - stats package), Multiple Factor Analysis - MFA (FactoMineR, factoextra) and another correlation matrix, cyanobacteria hits clustered by families were used. To create these graphs, 49 samples from the three main subregions were merged into 16 groups. The groups from Bulunkul included: Amorphous; Epiphytes/epiliths; Jelly-like; Multilayer hard; Multilayer soft; Non-layered and Non-layered beneath soil type (accordingly B_A, B_E, B_J, B_Mh, B_Ms, B_Nl, and B_Nlb). The ones from Karakul consisted of: Multilayer soft; Nostoc; Non-layered and Non-layered beneath soil type (K_Ms, K_N, K_Nl, and K_Nlb). Finally, the groups from Rangkul included: Multilayer hard; Multilayer soft; Nostoc; Non-layered and Non-layered beneath soil type (R_Mh, R_Ms, R_N, R_Nl, and R_Nlb). Additionally, package ggplot2 was used for creating violin plots, and Google spreadsheets were used for data visualization by bar graphs.

## 3. Results

### 3.1. Characterization of the studied subregions

The three subregions differed in terms of altitude, as well as environmental conditions of the studied reservoirs (Supplementary Table S1; Khomutovska et al., 2020). The highest elevated subregion was Karakul, as presented in Table 1. The water reservoirs studied in this subregion had on average 20°C, were slightly alkaline (pH 8–9), and highly varying in electrical conductivity (401–17,530 μS). They also had the highest mean nitrogen, calcium and magnesium concentrations and the lowest mean sodium concentration (6, 142, 662, and 58 mg/L respectively). The lowest elevated subregion, Rangkul, also had the lowest mean water temperatures in studied reservoirs (15°C), neutral to alkaline waters (pH 7–10), and the lowest mean electrical conductivity (1,711 μS). It was also the subregion with the lowest concentration of nutrients in the water, including mean total organic carbon (20 mg/L), nitrogen (3 mg/L), phosphorus (41 μg/L), potassium (23 mg/L), and calcium (64 mg/L). The Bulunkul subregion was slightly more elevated compared to Rangkul. It covered the largest area, was characterized by the most variable water bodies (including hot springs) and had the biggest number of sampling sites. In this subregion we noted the highest and the lowest temperature of a single reservoir (7 and 48°C), the highest and the lowest pH (6 and 10) and the highest and the lowest electrical conductivity (223 and 79,500 μS). Water in the reservoirs from which the mat samples were obtained had the highest mean organic carbon, sodium, potassium, and total phosphorus concentration as well as the lowest mean magnesium concentration (4,260, 28,800, and 1,340 mg/L, 1924 μg/L, and 155 mg/L accordingly).

**Table 1:** Lowest/highest (mean) values of altitudes, reservoir parameters and nutrient concentration.

	Bulunkul	Karakul	Rangkul
Number of samples	29	6	14
Altitude [m a.s.l.]	3,703/4,241 (3,830)	3,885/5,006 (4,179)	3,750/3,830 (3,776)
Temperature [°C]	7/48 (19)	10/29 (20)	9/24 (15)
pH	6/10 (8)	8/9 (8)	7/10 (8)
EC [μS]	223/79,500 (12,385)	401/17,530 (4,863)	402/4,286 (1,711)
C org [mg/L]	1/4,260 (226)	9/79 (29)	4/93 (20)
N tot [mg/L]	0.3/41 (6)	5/13 (8)	0.4/17 (3)
P tot [μg/L]*	0/1924 (15)	47/136 (75)	0/183 (41)
Na [mg/L]	2/28,800 (3,372)	10/170 (58)	14/370 (123)
K [mg/L]	0.7/1,340 (209)	4/400 (104)	3/94 (23)
Ca [mg/L]	3/330 (81)	28/470 (142)	36/180 (64)
Mg [mg/L]	2/155 (22)	17/2,541 (662)	24/491 (160)

*Because of the low amounts of P, it is described with a more precise unit.

**Figure 2:**
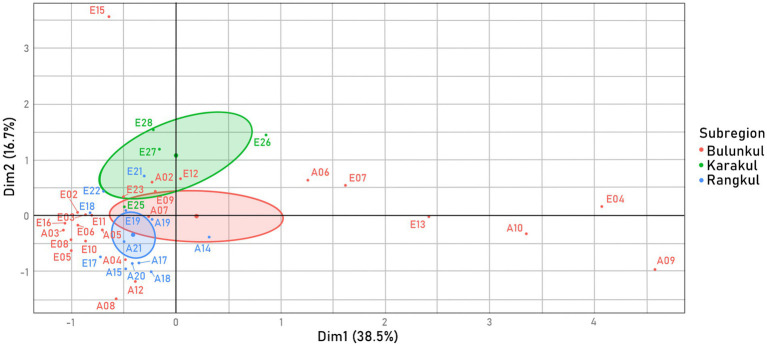
Variability of samples, MFA calculated from two factor groups: environmental parameters measured *in situ* (temperature, pH, salinity); and concentration of nutrients in reservoirs measured *ex situ* (total organic C, total N, total P, PO_4_^3−^, NH_3_^+^, NO_2_^−^ + NO_3_^−^, Na^+^, K^+^, Ca^+2^, Mg^+2^). The 41 samples were colored by a supplementary factor group: subregion, which was not included in the samples’ placement calculations.

The differences in parameters are reflected well by a Multiple Factor Analysis (MFA) that was calculated for 41 water samples. Samples without complete data were not included in this analysis. The graph with variability of the samples (Figure 2) shows the distribution of water samples based on the contribution of environmental parameters and nutrient concentration. Because temperature and salinity are factors which contributed the most to the position of the samples on the graph (temperature correlates positively with Dim2, and salinity correlates positively with Dim1–Supplementary Figure S2), samples from more saline reservoirs are dispersed toward the right side of the MFA, and the samples from warmer reservoirs toward the top of the graph. Reservoirs from Bulunkul have visibly the most diverse parameters, hence the extreme samples scattered along all edges of the graph. Although the majority of all the samples were grouped in the bottom left corner, samples from Karakul were placed above the Dim 1 axis, as they had the highest average water temperatures and moderate salinity. Water samples from the cooler and less saline reservoirs of the Rangkul subregion were condensed mostly to the left of the Dim 2 axis and shared a lot of similarities with the majority of samples from Bulunkul, which were characterized by less extreme conditions. Concentration of nutrients like NH_4_^+^, Mg^+2^, and PO_4_^3−^ had the lowest contribution to the variability of the samples.

### 3.2. Cyanobacteria diversity

Cyanobacteria was overall a dominant phylum in the Pamirian mats. The percentage of cyanobacterial ASVs varied between 1 and 80, with the mean of 35% in all samples. The other numerous phyla were Proteobacteria (28%), Bacteroidota (11%), Firmicutes (9%), Actinobacteriota (5%), Chloroflexi (3%), Planctomycetota (3%), and Verrucomicrobiota (2%) (Supplementary Figure S1). All ASVs identified as Cyanobacteria phylum can be divided into four groups: the first group is Oxyphotobacteria (Cyanobacteriia, according to Silva 138), which consists of 95.9% of all Cyanobacteria ASVs. The rest were identified as eukaryotic chlorophototrophs (chloroplasts) (3.7%), Vampirivibrionia (Melainabacteria) (0.3%) and Sericytochromatia (0.1%) (Soo et al., 2014, 2017). In this study we focused on photoautotrophic Oxyphotobacteria, since they were dominant in the samples and were observed using microscopic methods.

**Figure 3:**
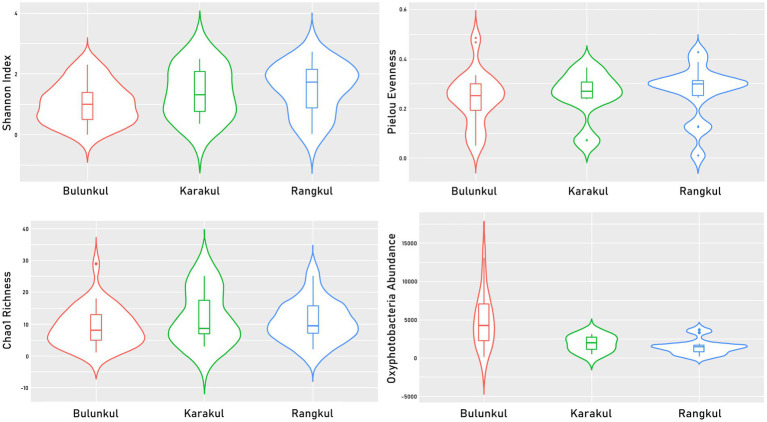
Violin plots presenting values of diversity indexes calculated for Oxyphotobacterial ASVs.

The diversity of Oxyphotobacteria was only slightly different in each subregion (Figure 3; Supplementary Table S2). In Bulunkul, the highest mean percentage of Oxyphotobacteria among other bacteria (44%) was found, as well as the highest mean abundance of Oxyphotobacterial ASVs (4913). The means of Shannon (1) and Pielou (0.2) indexes were the lowest on the other hand. The highest means of diversity indexes were noted in Rangkul (Shannon - 1.5, Pielou - 0.3), where we also found the lowest mean percentage of Oxyphotobacteria (19%), as well as the lowest mean abundance of Oxyphotobacteria (1463). In Karakul, the mean Pelou’s evenness was the same as in Rangkul (0.3), but the Shannon index was slightly lower and like the Bulunkul subregion (1). The means of Oxyphotobacteria percentage (22%) and abundance (1872) were in the same range as in Rangkul, but slightly higher.

**Figure 4:**
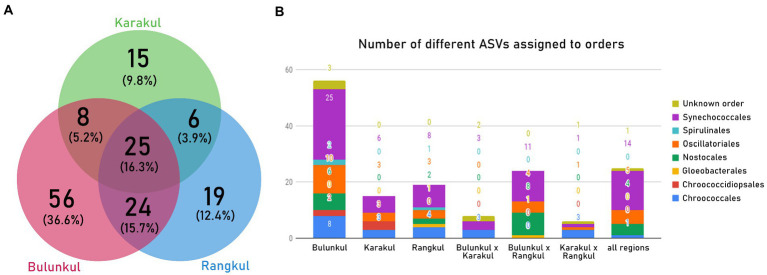
**(A)** Venn diagram showing the number of unique Oxyphotobacteria ASVs for each region as well as ones common for 2 or all regions. **(B)** ASVs from each part of the Venn diagram assigned to orders.

Some Oxyphotobacterial ASVs were found to be unique for each subregion and some of them were more universal occurring in two or three subregions. The number and percentage of unique and common sequence variants clustered to a species level (below referred as species) is presented in Figure 4A. Out of all 153 species, there were 63 shared between the subregions. Bulunkul and Rangkul shared 24 taxa, while Bulunkul and Karakul 8 species, and Karakul and Rangkul subregion 6 species only. There were 25 species common for all subregions with most of them belonging to the order Synechococcales (Figure 4B). The number of unique species corresponded to the number of samples collected from each subregion. In Bulunkul, where 29 mat samples were collected, 56 species were unique. These species belonged to orders Synechococcales, Oscillatoriales, Chroococcales, Nostocales, Spirulinales, and Chroococcidiopsidales. The remaining 3 species, each representing different sequences at the species level, could not be assigned to any order. In Rangkul, from 14 samples obtained, 19 unique species were noted. They were assigned to orders Synechococcales, Chroococcales, Oscillatoriales, Nostocales, and Gloeobacterales. In 6 samples from Karakul there were 15 unique species, belonging to orders Synechococcales, Oscillatoriales, Chroococcidiopsidales, and Chroococcales.

To minimize the influence of the number of samples on the number of unique and common sequence variants, we calculated their proportional distribution within each subregion (Table 2). Unique species in Bulunkul accounted for half of all species which were present in samples from this subregion. The share of species common for all subregions, as well as those common for Bulunkul and Rangkul, were both around 20%. The lowest share had the sequences common for Bulunkul and Karakul. In Rangkul and Karakul the unique ASVs accounted for slightly above 1/4th of the sequences with a higher percentage of sequences common for all three subregions. In Karakul the share of common sequences was the highest, accounting for almost half of all sequences found in this area.

**Table 2:** Distribution of unique and common ASVs for each region.

Region	Unique ASVs	ASVs common with Bulunkul	ASVs common with Rangkul	ASVs common with Karakul	ASVs common with other two subregions
Bulunkul	50%	–	21%	7%	22%
Rangkul	26%	32%	–	8%	34%
Karakul	28%	15%	11%	–	46%

### 3.3. Characterization of mat types based on their morphology

**Figure 5:**
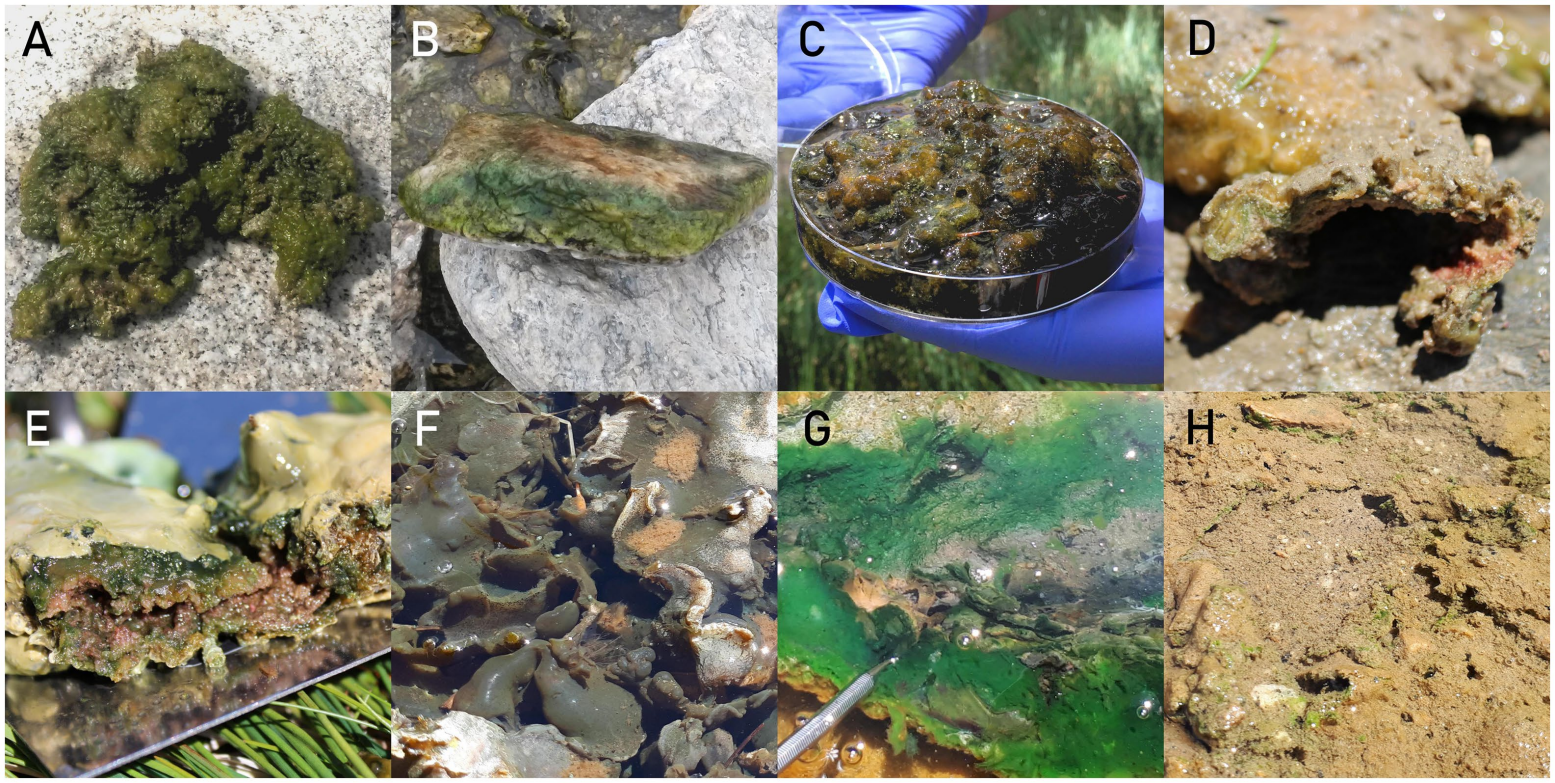
Photographs of mats taken *in situ*: **(A)** Amorphous type (placed on a rock near the pond). **(B)** Epiphytes/epiliths (growing on the surface of the rock taken from the stream). **(C)** Jelly-like (sample stored in a petri dish). **(D)** Multilayer hard (a cross-section of the mat). **(E)** Multilayer soft (a cross-section of the mat fold). **(F)** Nostoc type (mat floating on the surface of the reservoir). **(G)** Non-layered type (mat growing on the bottom of the stream, the edge is pushed away with a spatula, showing a green layer on the bottom of the mat). **(H)** Non-layered beneath soil (mat growing in a shallow pond, there are green splotches showing from under the sediment layer).

Microbial mats were classified into 8 distinct types based on their macro morphology (Figure 5): Amorphous (3 mats); Epiphytes/epiliths (2 mats); Jelly-like (2 mats); Multilayer hard (5 mats); Multilayer soft (13 mats); Nostoc type (4 mats); Non-layered type (17 mats) and Non-layered beneath soil (5 mats). This classification of mats in Eastern Pamir was published before by Khomutovska et al. (2020).

The amorphous type represents shapeless, coarse, considerably firm, and thick mats, composed of a mixture of filamentous cyanobacteria, *Nostoc* colonies and mosses. These mats are usually multicolored, from pale to dark green, to reddish and brown. The differently colored parts represent different organisms, but they do not form clear layers. Mats like this were found growing on the bottom of flat lake shores. The Jelly-like type is similar to the amorphous type but has a more loose and smooth consistency. Like the amorphous type, it also does not form layering and is similar in color. However, it has a visibly bigger contribution of Nostocales, with a large number of exopolysaccharides, creating a gelatinous form characteristic to this type. These mats grow on the bottom of shallow pools and between plants. When they are overgrowing, pillar or bubble-shaped forms can occur, which then detach from the part connected to the sediment and float on the surface.

The Epiphytes/epiliths type is a thin light to dark green film growing on rocks and higher plants. It is creating a smooth, rather thin layer made of filamentous cyanobacteria. The Multilayer hard type represents thick, firm, leather-like on the surface and undulating mats creating coral-like forms. The top layer is yellowish to dark brown, tough, and rigid, sometimes with visible round *Nostoc* colonies. The bottom layer is softer but still firm, pale green and pinkish with a visible large number of dense exopolysaccharides (EPS). These mats grow on the bottom and on the sides of reservoirs. The Multilayer soft type, on the other hand, is more delicate, sometimes even dissolving and is characterized by large amounts of EPS. They can be up to 20 cm thick, but after removing them from the water they collapse and shrink in thickness. They have three to four visible distinct layers. The top layer is very thin, gelatinous, pale green or pale orange. It consists of dead cyanobacteria cells, with high content of carotenoids and degraded chlorophyll, mixed with sediment. The middle part is of a dark green to emerald color. It is smooth, leather-like, and usually thin, composed of filamentous cyanobacteria. The third, red layer, is much thicker, nodular, sometimes accompanied by *Nostoc* and *Haematococcus* colonies embedded in thick EPS. Sometimes the fourth layer, below the red layer, is visible. It is green and consists of filamentous cyanobacteria. The mats of the Multilayer soft type grow at the bottom of reservoirs and between vascular plants, creating delicate folds that can detach and float on the surface of the water.

The Nostoc type mats have a characteristic uniform dark grayish-green color. They are firm, undulating and slightly coarse, formed by macroscopic, flat and large colonies with no layering, which is characteristic of *Nostoc commune*. They grow in very shallow pools and streams, usually floating near the surface and between vascular plants, or overgrowing wet soil.

The Non-layered type represents mats that are mainly made up of filamentous cyanobacteria. They are thin and delicate, slightly undulating but smooth in texture. The bright emerald green layer is usually located under the rusty reddish to pale brown, thin top layer. These mats can be found mostly in streams, but also pools and lake shores. They grow on the sediments, rocks and between plants, but can detach from the bottom sediments and float on the surface near reservoir borders. The last mat type, Non-layered beneath the soil, refers to mats growing under a thin layer of sediments, usually on a muddy bottom of shallow pools. They can vary from very thin to considerably thick (up to 0.5 cm), mostly dark green and undulating. The bottom part of the mat is coarser, whereas the part under the sediment layer is softer and more delicate. These mats can cover large areas of bottom sediments and are visible from afar as green speckles showing from under the sediments.

Multilayer soft and Non-layered type mats were the most diverse, based on microscopy (DESS) observations (Table 3). The first one was dominated by filamentous *Leptolyngbya*, *Lyngbya*, and *Phormidium* genera, as well as coccal colonial *Chlorogloea* and *Chroococcus*. They were accompanied by eukaryotic algae, mostly diatoms but also *Haematococcus* visible with the naked eye. In the Non-layered type most dominant genera were filamentous, like *Phormidium, Tychonema, Geitlerinema, Oscillatoria, Microcoleus*, and *Leptolyngbya*. In the Non-layered type mat sample (E15), growing in a hot spring along Gant River, a new filamentous genus, and species of cyanobacteria, *Hillbrichtia pamiri*a gen. et sp. nov., was found to dominate (Jasser et al., 2022). However, coccal *Chlorogloea* and *Aphanocapsa* genera, as well as heterocystous *Calothrix,* were also numerous in the Non-layered type mats. The Multilayer hard type was dominated by *Calothrix* and *Leptolyngbya* while Non-layered beneath soil by *Phormidium, Calothrix* and *Chlorogloea* genera. The least diverse was the Nostoc type, where only cyanobacteria from the *Nostoc* genus could be found in higher numbers. The remaining three types (Amorphous, Epiphytes/epiliths and Jelly-like) were also dominated by *Nostoc* or cyanobacteria from *Nostocaceae* family, along with other genera, like *Phormidium* and *Microcoleus* in the case of Amorphous type, *Pleurocapsa* in case of Epiphytes/epiliths or *Leptolyngbya* and *Chlorogloea* in case of the Jelly-like mat type.

**Table 3:** 0–1 matrix with Oxyphotobacteria orders identified molecularly (NGS) in each mat type compared with morphological identification (DESS).

Order/Mat type	Amorphous		Epiphytes/epiliths		Jelly-like		Multilayer hard		Multilayer soft		Nostoc		Non-layered		Non-layered beneath soil	
	NGS	DESS	NGS	DESS	NGS	DESS	NGS	DESS	NGS	DESS	NGS	DESS	NGS	DESS	NGS	DESS
Chroococcales	1	1	0	0	1	1	1	1	1	1	1	0	1	1	1	1
Chroococcidiopsidales	0	0	0	0	0	0	1	0	0	0	1	0	1	0	0	0
Gloeobacterales	0	0	0	0	0	0	1	0	0	0	0	0	1	0	1	0
Nostocales	1	1	1	1	1	1	1	1	1	1	1	1	1	1	1	1
Oscillatoriales	1	1	1	1	0	0	1	1	1	1	1	1	1	1	1	1
Pleurocapsales	0	0	0	1	0	0	0	0	0	0	0	0	0	0	0	0
Spirulinales	1	1	0	0	0	0	0	0	1	1	0	0	0	0	0	0
Synechococcales	1	0	1	0	1	1	1	1	1	1	1	0	1	1	1	1

### 3.4. Characterization of mat types based on V3-V4 region 16S rDNA

As in the microscopy observations, cyanobacterial orders which dominated in microbial mats according to molecular analysis were Synechococcales (with a total of 49% of reads in all mats), Oscillatoriales (22.3% of reads), and Nostocales (20.8% of reads). The remaining 7.9% consisted of Oxyphotobacteria orders Spirulinales, Gloeobacterales, Chroococcidiopsidales, Chroococcales, and Oxyphotobacterial reads that could not be assigned to any order, as well as Vampirivibrionia, Sericytochromatia, and chloroplasts.

**Figure 6:**
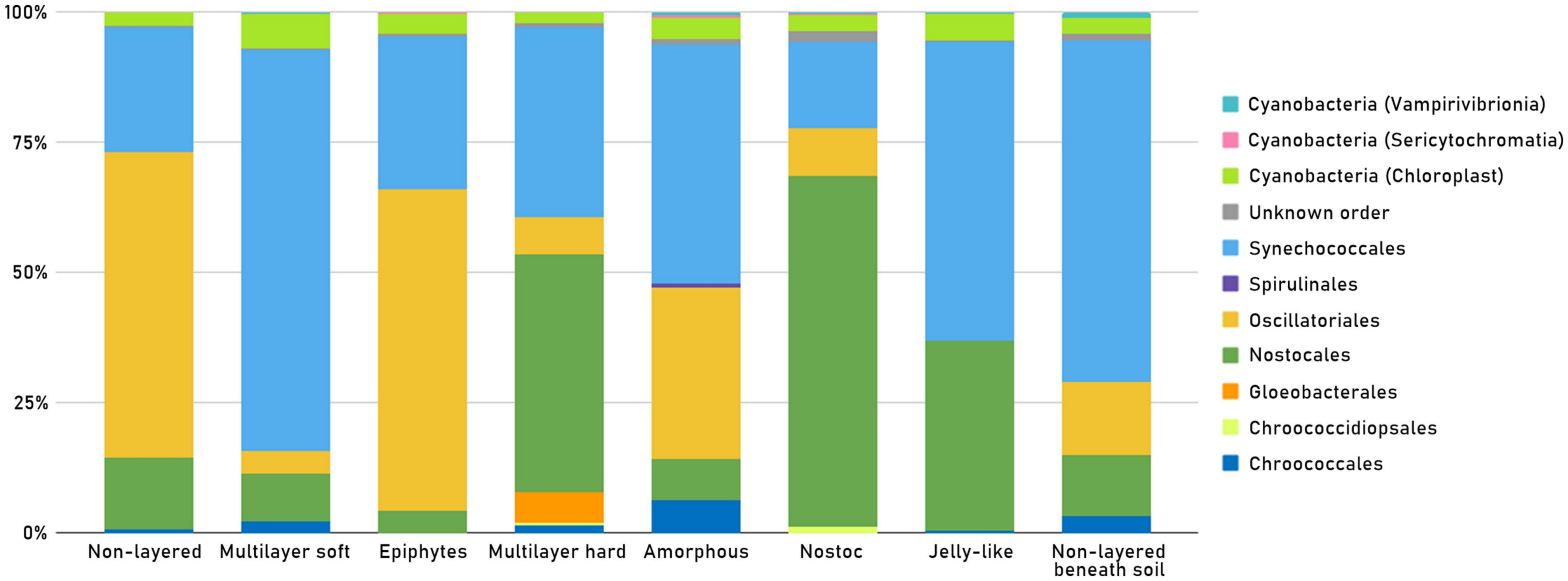
Presence of Oxyphotobacteria orders as well as other “Cyanobacteria” (Chloroplasts, Sericytochromatia, Vampirivibrionia) in each mat type, in all 51 samples.

The taxonomic structure of each of the mat types is shown in Figure 6. The Amorphous mats, which occurred in highly saline reservoirs, had the highest percentage of Chroococcales and Spirulinales within samples. However, Synechococcales together with Oscillatoriales, accounted for almost 80% of all cyanobacterial reads in this type of mat. The dominant taxa in Amorphous mats (samples A10 and E13, with salinity 45 and 39 ppt accordingly) were *Leptolyngbya* spp. (36% of all Oxyphotobacteria), accompanied by *Oscillatoria acuminata* (23%), *Jaaginema* (16%), and *Coleofasciculus* (10%).

It was also the mat type with the highest percentage of Sericytochromatia. Epiphytes/epiliths had the highest percentage of Oscillatoriales and the lowest contribution of Nostocales. The Jelly-like type was mostly composed of Synechococcales and Nostocales with no representation of the Oscillatoriales order. The Non-layered type, on the other hand, had one of the highest percentages of Oscillatoriales within samples. The Non-layered beneath soil was a mat type with one of the highest percentages of Synechococcales, as well as the highest percentage of Vampirivibrionia. One of the Non-layered beneath soil mats was sample E04, collected from a hypersaline reservoir, where the most dominant taxa were *Leptolyngbya* spp., constituting 48% of Oxyphotobacterial taxa. The other 26% were Oscillatoriales (*Geitlerinema* sp. and *Oscillatoria acuminata*), and the 25% were Nostocales (*Calothrix* spp., *Nostoc* sp. and *Nodularia spumigena*). In the Nostoc type, the dominant order was Nostocales. The highest percentage of Chroococcidiopsidales was also present in this mat type. The Multilayer hard type had the highest percentage of Gloeobacterales, but it was mostly composed of Nostocales and Synechococcales. The highest percentage of Synechococcales and of chloroplasts was noted in the Multilayer soft mat type.

We also compared the presence of Oxyphotobacteria orders in all mat types, identified molecularly and morphologically (Table 3). Orders like Nostocales and Synechococcales were found in all mat types by NGS, but only Nostocales were also identified morphologically in all types. Orders Chroococcidiopsidales and Gloeobacterales were found by NGS in four of the types but were not found under a microscope. Order Spirulinales was identified in two mat types by both NGS and morphological analysis. Order Pleurocapsales was found in one mat type under a microscope but was not identified by NGS. 29 out of 40 identifications of certain orders in all mat types carried out by NGS analysis were confirmed by morphological analysis. In Jelly-like and Multilayer soft types, results obtained using both techniques overlapped fully. Also at the genus level NGS methods allowed for identifying many more taxa than based on morphology. The highest number of genera was found in Non-layered and Multilayer soft types, 28 and 23 genera, respectively (Supplementary Table S3).

**Figure 7:**
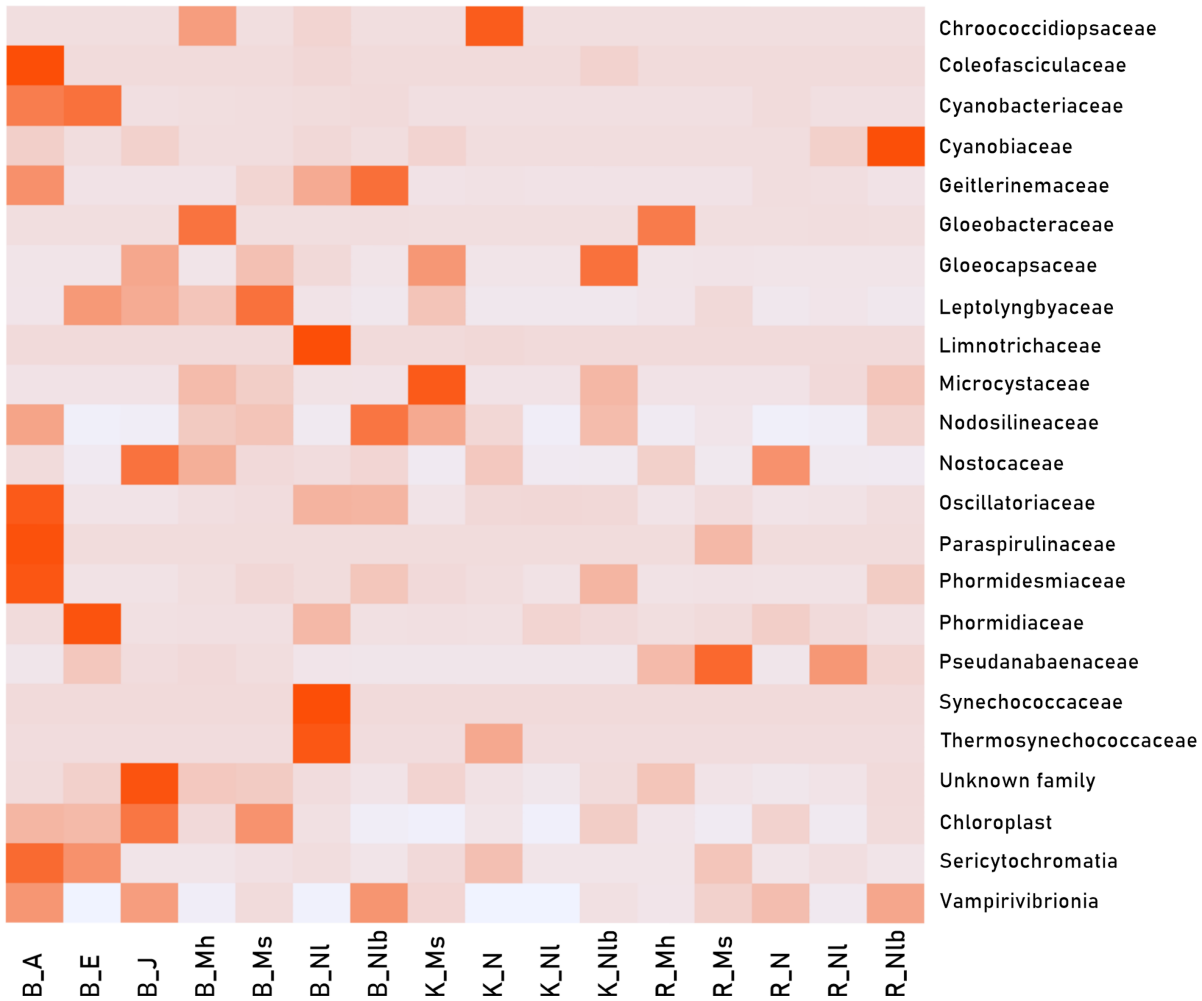
Heat map calculated from Oxyphotobacteria family reads and other “Cyanobacteria” (Chloroplasts, Sericytochromatia, Vampirivibrionia) in 49 samples merged into 16 representative groups: B_A - Bulunkul Amorphous; B_E - Bulunkul Epiphytes/epiliths; B_J - Bulunkul Jelly-like; B_Mh - Bulunkul Multilayer hard; B_Ms - Bulunkul Multilayer soft; B_Nl - Bulunkul Non-layered; B_Nlb - Bulunkul Non-layered beneath soil; K_Ms - Karakul Multilayer soft; K_N - Karakul Nostoc; K_Nl - Karakul Non-layered; K_Nlb - Karakul Non-layered beneath soil; R_Mh - Rangkul Multilayer hard; R_Ms - Rangkul Multilayer soft; R_N - Rangkul Nostoc; R_Nl - Rangkul Non-layered; R_Nlb - Rangkul Non-layered beneath soil. Dark red shades represent a high number of sequences and light gray color represents no sequences.

To show a more precise cyanobacterial structure of the different types of mats concerning their geographical location, samples merged into groups were used Figure 7. shows the percentage ratio of Oxyphotobacterial families as well as other Cyanobacteria (Chloroplasts, Sericytochromatia, Vampirivibrionia) in the representative groups. The much higher overall abundance of Cyanobacteria in Bulunkul, in comparison to other subregions, is clearly visible on the graph. The highest numbers of Chloroplasts, Sericytochromatia, and Vampirivibrionia could also be observed in this subregion. The Amorphous, Epiphytesepiliths and Jelly-like (B_A, B_E, B_J) mats were present only in Bulunkul and their structure corresponds to the one previously described in Figure 6. There were however mat types that could be found in two subregions: Multilayered hard mats in Bulunkul and Rangkul (B_Mh, R_Mh); and Nostoc type in Karakul and Rangkul (K_N, R_N). The Multilayered hard mats were characteristic for their high number of sequences classified as *Gloeobacteraceae*. The Nostoc type was not so alike with visible domination of *Chroococcidiosidaceae* sequences in Karakul and absence of them in Rangkul. The *Nostocaceae* family was still prevalent in both subregions but more numerous in Rangkul. The Multilayered soft (B_Ms, K_Ms, R_Ms), Non-layered (B_Nl, K_Nl, R_Nl) and Non-layered beneath soil (B_Nlb, K_Nlb, R_Nlb) mat types were present in all three subregions. In the Multilayer soft mats, the majority of dominant families belonged to the Synechococcales order (*Leptolyngbyaceae*, *Nodosilineaceae*, *Pseudanabenaceae*) and there was also a high number of Chroococcales (*Microcystaceae*, *Gloeocapsaceae*). When it comes to the Non-layered type, there was a high number of *Synechococcaceae*, *Thermosynechococcaceae* and *Limnotrichaceae* in Bulunkul but in other two subregions a much lower diversity was observed. Non-layered beneath soil type differed the most between the subregions. *Geitlerinemaceae* and *Nodosilineaceae* families were dominant in Bulunkul, *Gloeocapsaceae* in Karakul, and *Cyanobiaceae* in Rangkul.

**Figure 8:**
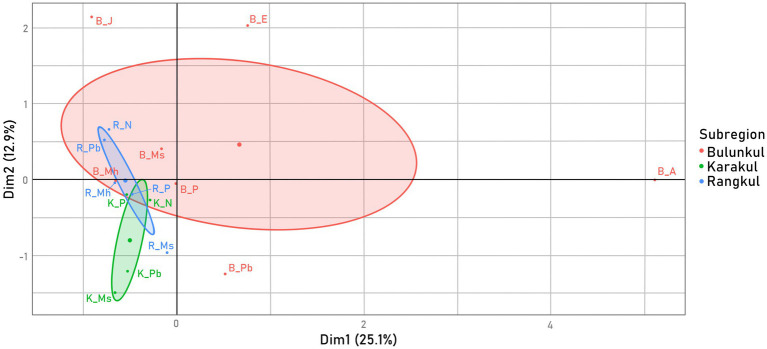
Variability of samples - MFA calculated from two factor groups of Cyanobacteria family reads from the representative groups: Oxyphotobacteria and other Cyanobacteria, colored by a supplementary factor group: subregion, which was not included in the samples’ placement calculations.

The same data was used in Figure 8, where representative groups were compared on an MFA graph. The biggest contribution to Dimension 1 was from the Oxyphotobacteria families such as Coleofasciculaceae, Paraspirulinaceae, Oscillatoriaceae, Phormidesmiaceae as well as from Vampirivibrionia (unspecified family), Vampirivibrionia (Gastranaerophilales) and Sericytochromatia (Supplementary Figure S3). For Dimension 2, the biggest contribution had eukaryotic chlorophototrophs (chloroplasts), Vampirivibrionia (Vampirivibrionales), and Vampirivibrionia (Obscuribacterales). Overall, groups from Bulunkul differed the most from the others, but the placement of some of them overlapped with the placement of groups from Rangkul. Groups from Karakul were much more distinct. Mats from Bulunkul, B_J, B_E and B_A had the highest abundance of cyanobacteria, therefore their placement on the edges of the graph is more pronounced. Although B_Nl did not have as low an abundance as K_Nl and R_Nl did, the three groups representing Non-layered type mats were located close to each other, near Dimension 2 axis. The other mat type, in which representative groups were placed a short distance from each other, was a Multilayer hard type.

### 3.5. Relations between the taxonomic composition of microorganisms and the physical and chemical parameters of water

**Figure 9:**
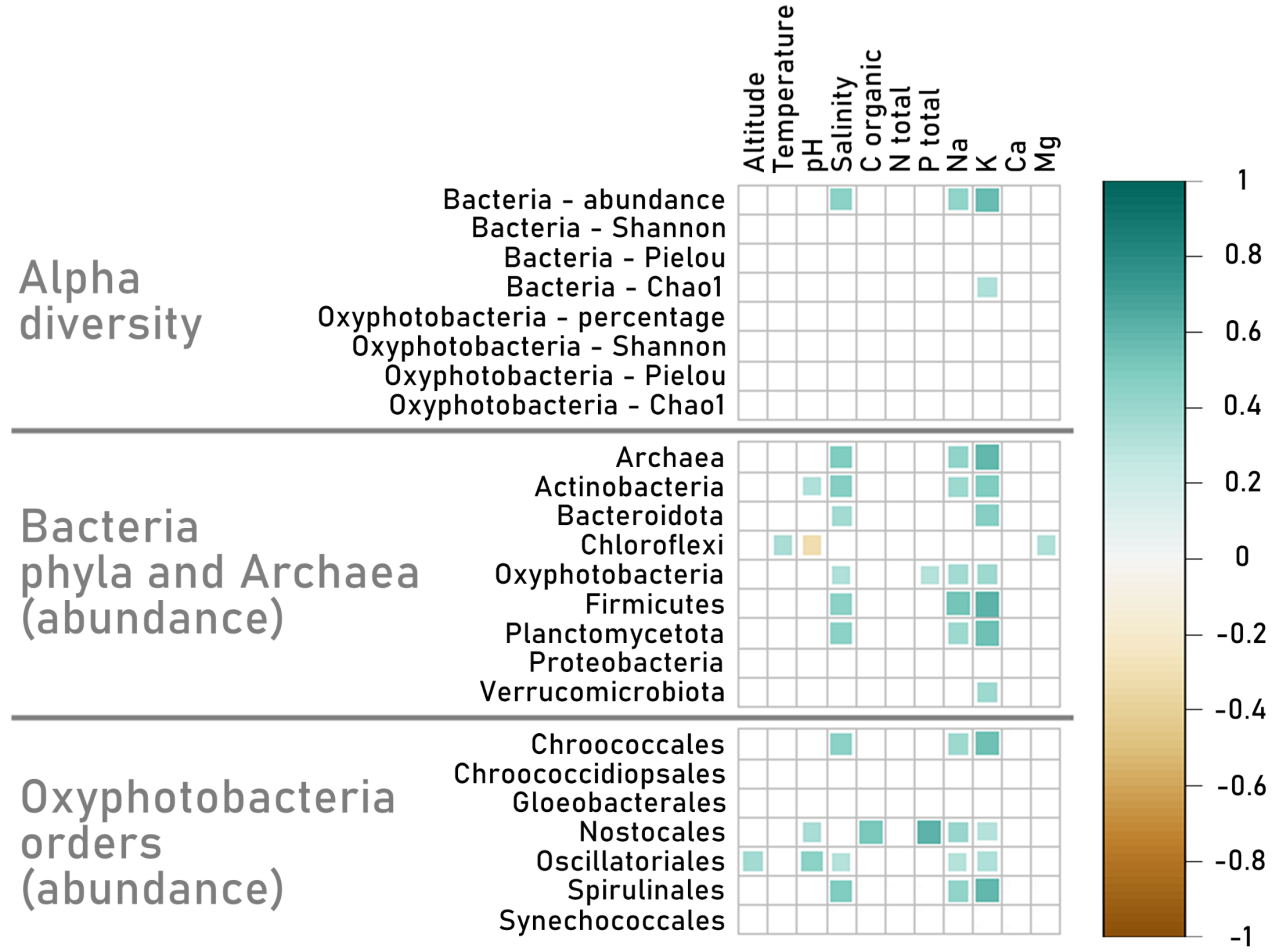
Matrix of Spearman’s rank correlations between environmental parameters (altitude, temperature, pH, salinity, total organic C, total N, total P, PO_4_^3−^, NH_3_^+^, Na^+^, K^+^, Ca^+2^, Mg^+2^) and alpha diversity (Shannon, Pielou and Chao1 indexes for Bacterial phyla and Oxyphotobacterial orders, as well as Bacterial abundance and Oxyphotobacterial percentage in samples) and abundance of Bacteria phyla, Archaea, and Oxyphotobacterial orders based on the number of reads on the ASV level. The graph is showing only statistically significant results (*p* < 0.05). The Bacteria phyla used in this matrix are those whose abundance results in min. 1% of all Bacterial abundance. Nine samples were excluded because of the lack of a complete set of environmental data.

Correlations between bacterial diversity and the relative abundance of certain phyla or Oxyphotobacterial orders with the abiotic data turned out to be statistically significant in some cases (Figure 9). First of all, the salinity as well as sodium and potassium concentration, correlated positively with the total number of Bacterial ASVs (Bacteria - abundance). These factors correlated the most with the abundance of some bacterial phyla, like Oxyphotobacteria, Actinobacteriota, Bacteroidota, Firmicutes, and Planctomycetota. In addition, the potassium concentration correlated with Bacterial Chao 1 index (0.3). The abundance of Chloroflexi however was related more to higher temperatures (0.4), lower pH (−0.3) and higher magnesium (0.3) concentration in water. Oxyphotobacteria showed an additional tendency to be more abundant in waters with higher total phosphorus concentration (0.3). It may be a result of the high percentage of Nostocales in the samples, because they showed a high positive correlation with some chemical parameters i. e. total phosphorus concentration (0.6), along with pH (0.4), organic carbon (0.5), sodium ion (0.4) and potassium ion (0.3) concentrations. The other dominant order, Oscillatoriales, correlated positively with altitude (0.4), pH (0.5), salinity (0.3), sodium (0.3) and potassium (0.3). These last three factors also correlated positively with Chroococcales and Spirulinales, however, these orders were not prevalent in the samples. Interestingly the abundance of the most dominant order, constituting almost 50% of all Oxyphotobacterial reads, Synechococcales, did not correlate with any abiotic factors.

**Figure 10:**
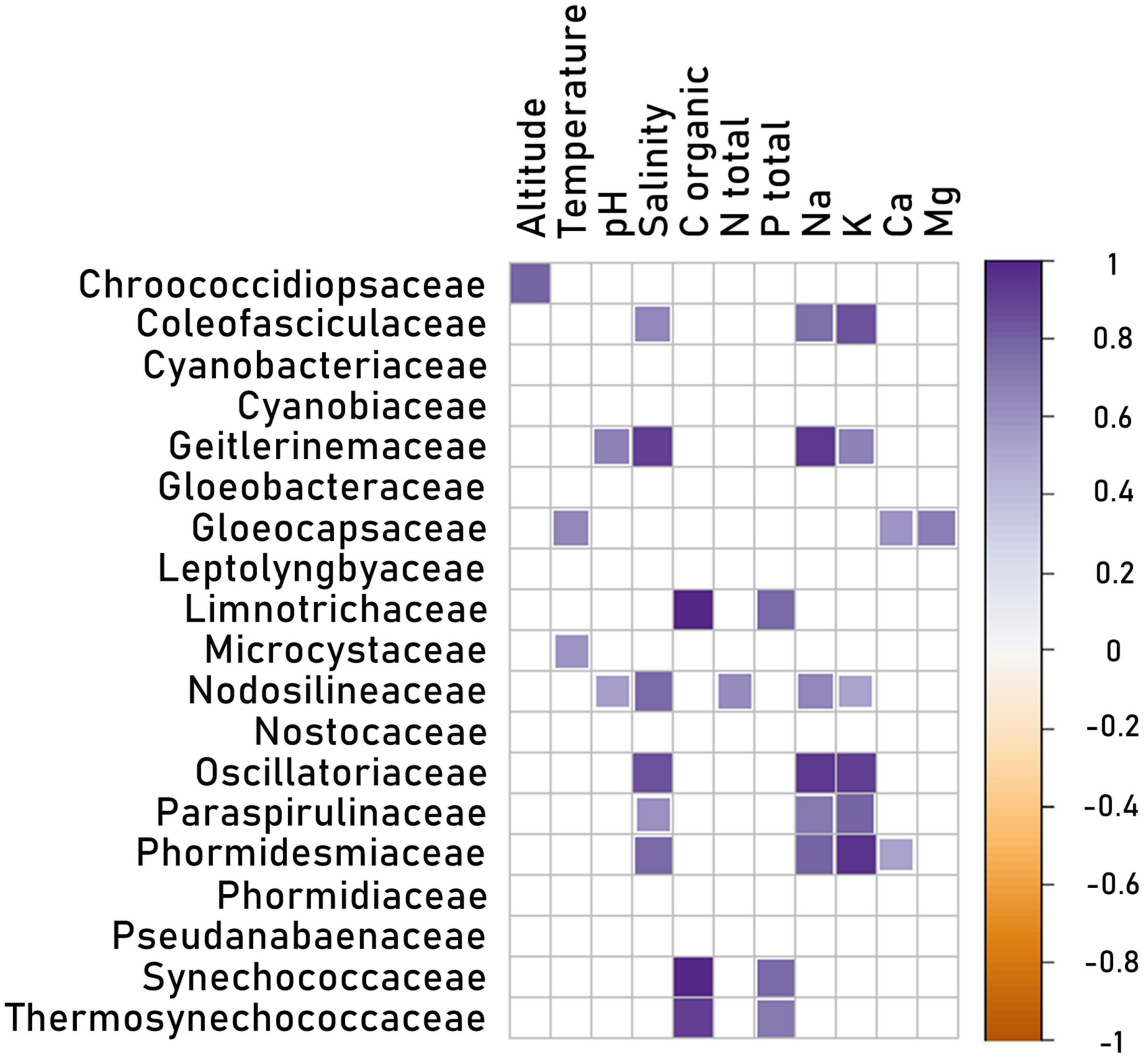
Matrix of Spearman’s rank correlations for environmental parameters (altitude, temperature, pH, salinity, total organic C, total N, total P, PO_4_^3−^, NH_3_^+^, Na^+^, K^+^, Ca^+2^, Mg^+2^) and Cyanobacteria family reads in 48 samples merged into 15 representative groups (group K_Nl was excluded because of lack of environmental data).

The abiotic parameters were also studied with the abundance of Oxyphotobacterial families in the representative groups of different mat types in different subregions (Figure 10). There was a positive correlation between certain families and altitude (Chroococcidiopsaceae), temperature (Gloeocapsaceae and Microcystaceae), and pH (Geitlerinemaceae and Nodosilineaceae). The abundance of some families correlated with the concentration of organic carbon (with Limnotrichaceae, Synechococcaceae and Thermosynechococcaceae), total nitrogen (with Nodosilineaceae), total phosphorus (with Limnotrichaceae, Synechococcaceae and Thermosynechococcaceae), calcium (with Gloeocapsaceae and Phormidesmiaceae), and magnesium (with Gloeocapsaceae). However, most families, such as Coleofasciculaceae, Geitlerinemaceae, Nodosilineaceae, Oscillatoriaceae, Paraspirulinaceae, and Phormidesmiaceae, correlated positively with salinity. The correlations with salinity were also coinciding with positive correlations with sodium and potassium. Additionally, if the families correlated with other parameters the correlation with salinity or Na i K was more pronounced.

## 4. Discussion

### 4.1. Morphological and molecular identification of cyanobacteria and other bacteria forming microbial mats

In this paper, we describe the diversity and structure of microorganisms in very diverse photosynthetic microbial mats overgrowing small water bodies in the high-altitude plateau of the Eastern Pamir Mountains. In general, authors divided microbial mats on the basis of environmental characteristics, such as the hypersaline, coastal, oligotrophic, psychrophile, hot springs, and acid microbial mats (Prieto-Barajas et al., 2018). Others divided the mats according to their macromorphology (Feder et al., 2013). In our study, having a very high morphological variety of mats, we divided them according to their macromorphology into eight types.

Ever since the introduction of molecular methods of the identification of microorganisms they have become an integral part of almost every study giving new insight into the composition and structure of microbial communities. These methods are also widely used in cyanobacteria research in which they complement and sometimes even prevail over morphological studies. That’s because differentiation based on morphology can lead to many possible mistakes in identification for example because of cryptic species and genera (Komárek, 2018). By comparing the results of the next generation sequencing (NGS) analysis and the light microscopy analysis of the preserved environmental samples (Table 3) we observed that there was a greater variety of cyanobacterial orders identified with the molecular methods. In our study the great majority of cyanobacteria which were unnoticed *via* light microscopy belonged to the coccal colonial and unicellular genera and families, and were identified by NGS in only small numbers. The only case when the order was observed in the environmental sample but was not identified by NGS was Pleurocapsales in the Epiphytes/epiliths mat type. The reason for that could be that despite several isolation attempts the Pleurocapsales DNA did not isolate properly because of thick sheaths around the cells. Similarly, we have observed the inconsistencies in the results of NGS analysis of Nostoc type mats (Figure 7). Two samples of Non-layered beneath soil mats from Karakul (E25 and E28), which on a macro-scale were dominated by *Nostoc* colonies, had only 14% of Oxyphotobacterial sequences classified to Nostocales.

The bacterial structure varied between samples (Supplementary Figure S1). Most of them were dominated by Cyanobacteria followed by Proteobacteria, and Bacteroidota, similarly to the surface layer of mats from Shark Bay in Australia (Wong et al., 2015). Domination of cyanobacteria was also noted in thermal spring mats studied with DGGE fingerprints (Portillo et al., 2009; Coman et al., 2013; Mackenzie et al., 2013) but the contribution of other bacterial phyla was different than in our hot spring sample. In general, however, the dominance of Cyanobacteria over other bacterial phyla in the microbial mats seems to be characteristic of hypersaline environments (Bolhuis et al., 2014; Prieto-Barajas et al., 2018). In the case of microbial mats in Eastern Pamir the highest percentage of Cyanobacteria was noted in oligosaline reservoirs though also considerably high in saline ones. This suggests that in Eastern Pamir microbial mats with pronounced cyanobacteria domination appear to cover a great range of environmental variability at least in the case of salinity.

Our NGS analysis shows that more than 92% of cyanobacterial sequences in the mats belonged to the orders Synechococcales, Oscillatoriales, and Nostocales. A vast majority of them were filamentous, which is very typical for mat-forming cyanobacteria (Stal, 2012). The warmest reservoirs were dominated by Synechococcales and Oscillatoriales, which is partially compatible with literature. The thermophilic microbial mats with temperature exceeding 55°C are known to be composed mostly of unicellular *Synechococcus* species (Prieto-Barajas et al., 2018). However, in the Pamirian hot spring (E15), with temperature around 50°C, a filamentous *H. pamiria* gen. Nov. sp. nov. belonging to the subclass Oscillatoriophycidae (Jasser et al., 2022), was the most abundant cyanobacterium. When it comes to the psychrophilic mats, Oscillatoriales and Nostocales are the most common cyanobacterial orders, according to the literature (Taton et al., 2003; Prieto-Barajas et al., 2018). In our samples however, Nostocales prevailed only in temperate waters, while the coldest reservoirs were dominated only by Oscillatoriales and filamentous Synechococcales, mostly *Leptolyngbya* species.

*Leptolyngby*a was also the genus that thrived in the hypersaline pond, constituting almost half of all cyanobacterial reads. Other highly saline mats in this study were also characterized by high contribution of *Leptolyngbya* with smaller contribution of *O. acuminata*, *Jaaginema* and *Coleofasciculus*. Prieto-Barajas et al. (2018) characterizing hypersaline mats from various regions in the world have found that *Microcoleus, Oscillatoria, Phormidium*, and *Lyngbya* were the predominant genera accompanied by unicellular *Gloeocapsa*, *Synechococcus* and *Cyanothece* and sometimes heterocystous *Calothrix*. In the other study on hypersaline mats (Abed et al., 2011) the main mat-building cyanobacterium was *Microcoleus chthonoplastes* (*Coleofsciculus chtonoplastes*). The occurrence of *Leptolyngbya* in hypersaline mats was noted by other authors, but contribution of this genus was low (Feder et al., 2013; Wong et al., 2015; Ramos et al., 2017). Thus, the dominance of *Leptolyngbya* in the hypersaline and sub-dominance in other highly saline mats in Pamir seems to be quite a unique feature distinguishing them from others observed in hypersaline environments.

The differences in the dominant taxa between our results and the literature may arise from various reasons. One of them is the fact that the mat-forming cyanobacteria are still poorly studied in terms of molecular data. Most sequences in the databases belong to planktic cyanobacteria and there are much fewer sources to obtain the information about benthic microorganisms from. Research recently published by Salmaso et al. (2022) on a coverage of 16S rRNA sequences from environments of considerably well-studied alpine lakes and rivers showed that 30% of planktic and 60% of biofilm sequences had no close match in public databases. Comparing microscopic identifications with molecular data the authors found the gap even larger. We faced the problem of a lack of highly similar sequences while studying endolithic and soil-inhabiting communities from Eastern Pamir (Khomutovska et al., 2021a,b). Also, in the present study we obtained many sequences that were identified only to Oxyphotobacteria class. The results suggest that many new taxa of cyanobacteria from mats in diverse small water bodies in Pamir are yet to be discovered.

The disparities between the morphological observations and molecular results were even more pronounced after identification data automatically generated from the SILVA database on the higher taxonomic ranks. There were no sequences identified as Nostocales or Oscillatoriales and only very few assigned to Synechococcales order. This type of structure contradicted even our earliest macro-scale observations, e.g., the identification of mats that were mostly composed of spherical *Nostoc* sp. colonies visible with the naked eye. Only the verification of query sequences using a phylogeny-based placement method allowed for more accurate identification to higher taxonomic ranks which was presented and discussed in this paper. Unfortunately, even though we were able to assess cyanobacterial structure of the mats on the order level, it was not possible to unequivocally describe genera and species based solely on amplification of 16S rRNA V3-V4 hypervariable regions. To obtain identification on these levels, analyses of the metagenomes and filtered whole 16S rRNA sequences could be a solution. However, there is still a need for reference strains and sequences from various unique environments including extreme habitats. Another reason for some inadequacy in the composition and structure of cyanobacterial community based on sequencing could arise from the way samples were prepared for further analyses, which was drying them in open air. Wind and dust could be sources of contamination of the samples. The additional subsamples for DNA isolation, which were fixed with DESS on the spot just after sampling, to some extent reduced the environmental source of contamination. Unfortunately, we have not used samples containing only DESS as a blank, thus the fixative could also contain some nonspecific sequences that could influence our results. Nevertheless, results of cyanobacterial taxonomic composition and structure obtained in this study present communities that are very different from each other, with various dominants and subdominants which was verified by phylogenetic placement.

It is also worth mentioning that in several of our samples there were 4 types of ASVs belonging to class Sericytochromatia, and 8 belonging to Vampirivibrionia (Melainabacteria) identified. These are non-photosynthetic cyanobacteria, sister clades to Oxypthotobacteria, that supposedly retained a simpler metabolism from the ancestors of all Cyanobacteria phylum (Soo et al., 2014, 2017). There is not much information about these organisms yet, but we can now confirm their occurrence in the microbial mats from the cold desert of Eastern Pamir.

### 4.2. Influence of location and water parameters on taxonomic composition

The microbial mat communities in the Eastern Pamir Mts. occurred in multiple different types of reservoirs and under the impact of highly variable environmental factors. These miscellaneous conditions resulted in the development of unique structures and taxonomic composition described above. However, even though the conditions of studied reservoirs varied, there was a pattern in their similarities within each subregion. Salinity and temperature were the most important environmental parameters influencing the variability of the sampling sites, although concentrations of nutrients, organic carbon, pH and to some extent the altitude varied between them.

In the previous study conducted in Pamir, concerning benthic biofilms in small water bodies no clear differences between samples from the Bulunkul and Rangkul area were observed (Jasser et al., 2019). According to that study, the inocula of cyanobacterial benthic communities from the same area differed significantly, whereas the ones from reservoirs with similar chemical composition of water, especially salinity, were more alike. In the case of endolithic cyanobacteria in Eastern Pamir studied in the same three remote locations, there was also no pattern of distribution of microorganisms based on geographic distance (Khomutovska et al., 2021a,b). However, in biological soil crust (BSC) with which endolithic communities were compared, bacterial structure was influenced more visibly by geographical location. Cyanobacteria living in the upper layers of soil are more dependent on precipitation and water availability, which can influence their diversity more than the chemical properties of soil (Büdel et al., 2008; Hagemann et al., 2015), and which was confirmed by our results. In contrast to the BSC, the communities of benthic, mat-forming cyanobacteria generally have an abundance of water. Thus, the variability of these communities seems to relate to the fluctuations of chemical and physical parameters of water.

Analyzing cyanobacterial mats in relation to geographical distance, we have found some indications of differences in the diversity parameters, presence of unique and common sequences as well as distribution of families in representative types of microbial mats. The distribution of reads at the family level for representative groups showed a similar pattern as the distribution of microenvironmental parameters in these areas (Figures 2, 8). In both cases, samples, or representative groups from Bulunkul were the most diverse and overlapped with the ones from Rangkul while samples and representative groups from the Karakul area formed a mostly distinct and separate cluster. This might suggest that subregions differed to such an extent that it influenced the mat-forming cyanobacteria. However, these patterns characteristic for the tree locations, may still be connected with microenvironmental differences between the reservoirs. Thus, we have no definite proof that geographical distance and isolation between sampling areas directly influenced the diversity, composition, and structure of studied cyanobacterial mats. To further verify the first hypothesis, we analyzed the influence of the physical and chemical water parameters on the microbial mat community. We found significant correlations between several water parameters and Oxyphotobacterial structure.

Studies on planktic bacterial communities revealed that they are highly influenced by environmental conditions. Both abundance of bacteria and the taxonomic composition of the consortia can be correlated with pH, dissolved oxygen, or trophic state of the water (Chopyk et al., 2018; Kiersztyn et al., 2019). The composition, structure and biomass of phytoplankton are highly correlated with nutrient concentration, pH, temperature and also salinity (Reynolds, 1998; Jia et al., 2022). Interestingly, higher salinity was found to influence the germination of akinetes as well as the toxicity of *N. spumigena*, regardless of nutrient and temperature changes (Silveira and Odebrecht, 2019). In microbial communities from lake sediments, salinity (along with pH) was found to influence bacterial diversity and structure the most (Yang et al., 2016). Bolhuis et al. (2014) studying microbial mats in the salinity gradient on the shores of Schiermonnikoog Island found that salinity was the major driver shaping the mat communities manifesting in cyanobacteria domination in primary production in brackish environment, diatoms in marine and cyanobacteria and green algae in freshwater part. The authors noted also that cyanobacteria exhibited the highest Shannon index in the brackish environment. Also in desert soil, salinity played a big role in shaping the microbial communities, since it correlated negatively with their abundance and diversity and was the main factor that, according to the authors, determined their structure (Zhang et al., 2019).

Salinity and temperature, which seemed to be the most important variables defining the variability of the studied small water bodies, influence the microorganisms in very specific ways. High salt concentration creates stressful conditions which require special physiological adaptations. Some Bacteria and Archaea accumulate high concentrations of ions K^+^ and Cl^−^ in cells to balance those from the outside of the cell. Cyanobacteria however (like most bacteria) to withstand the osmotic stress accumulate organic osmotic solutes. In less saline environments cyanobacteria store disaccharides (sucrose and trehalose) in their cells, in more saline ones, they accumulate glucosylglycerol in cytoplasm, and glycine betaine in moderately saline and hypersaline (Oren, 2015). High water temperature also requires various physiological adaptations, such as producing antioxidants, or inducing various defense genes, for example genes responsible for biosynthesis of heat shock proteins or desaturases, enzymes responsible for fatty acids desaturations in membranes (Ismaiel and Piercey-Normore, 2021). Not only induction of certain genes but also their number, as in the case of HSPs genes, is connected with the ability of cyanobacterial species to grow in stressful conditions, for example high temperature environments (Jasser et al., 2022). In this way we can say that salinity or temperature may influence the composition and structure of cyanobacterial communities by favoring the growth of organisms that can thrive in such conditions.

In the present study, salinity was also a prevalent factor correlating with microbial composition (Figure 9). The higher overall salinity along with the sodium and potassium content, the higher abundance of Bacteria was observed. It also coincided with the higher abundance of Oxyphotobacteria in samples, including one of the dominating orders, Oscillatoriales. A similar pattern was observed when comparing the concentration of macro and microelements in water with the abundance of Oxyphotobacterial families suggesting that salinity and the sodium and potassium influence the taxonomic composition of mats to a high degree (Figure 10). Jasser et al. (2019) found out in earlier research that salinity, and to some extent, temperatures were key factors influencing the composition of benthic cyanobacteria inoculum, but the structure must have been shaped by other factors as well. In the present study another factor that was associated with cyanobacterial communities was pH, which correlated positively with Nostocales and Oscillatoriales. Also, organic carbon and total phosphorus concentrations seemed to influence some Oxyphotobacteria.

Similarly, to the data found in the literature, cyanobacterial mats in the present study (as presented above) were influenced mostly by salinity, but contrary to the case of the desert soil Oxyphotobacteria (Zhang et al., 2019), their abundance increased with higher salinity. This verifies positively the second hypothesis, that salinity affects the microbial structure the most of all the factors. The number of samples analyzed in this study as well as the wide range of salinity additionally support the result. Recently, other authors found a positive correlation between salinity and abundance and structure of cyanobacteria pointing to an unexpected abundance of heterocystous cyanobacteria in hypersaline microbial mats (Campbell et al., 2022).

In conclusion, the cold mountain desert in Eastern Pamir Mts hosts numerous water bodies which are characterized by diverse chemical and physical water features. In this environment, very rich and variable cyanobacterial mats overgrow bottom sediments and shores of the water bodies. These mats were classified into eight types based on their macromorphology and studied for the composition and structure of cyanobacterial communities. The comparison of morphological and molecular analyses exhibited disparities between identification of cyanobacteria with these methods and demonstrated the role of NGS in revealing the composition of these communities. The study also exposed the lack of close matches for many sequences from these environments in databases. There was a high percentage of cyanobacteria in the bacterial composition of the mats throughout a high range of salinity. Synechococcales proved to be the order that dominated most of the mat types with Oscillatoriales and Nostocales following suit. We also found many unidentified sequences of Oxyphotobacteria, with, at present, one new species and genus, as well as some sequences representing Sericitochromatia and Vampirivibrionia. Given our results, we can support the first hypothesis that microenvironmental factors are more important than geographical distance in shaping the diversity of cyanobacterial communities in Eastern Pamir. Salinity and concentration of Na and K seemed to be the key microenvironmental factors influencing the diversity of mats at the family level, which supports the second hypothesis.

## Data availability statement

The datasets presented in this study can be found in online repositories. The names of the repository/repositories and accession number(s) can be found in the article/Supplementary material.

## Author contributions

IJ and JK: conceptualization. IJ: study design and supervision. MS and NK: data curation. MS, NK, and ŁŁ: formal analysis (molecular, statistical and bioinformatic analysis) and investigation. NK, JK, MS-M, TN, and IJ: sampling area selection, field works, and sampling. MS: writing—original draft preparation (cyanobacteria isolation and cultivation, morphological analyses of mats and cyanobacteria) and visualization. IJ, NK, and MS-M: writing—review and editing. TN: resources. IJ and MS-M: funding acquisition. All authors have read and agreed to the published version of the manuscript.

## Funding

This work was supported by the National Science Centre (Grant 2015/19/B/NZ9/00473 and 2013/09/B/ST10/01662).

## Conflict of interest

The authors declare that the research was conducted in the absence of any commercial or financial relationships that could be construed as a potential conflict of interest.

## Publisher’s note

All claims expressed in this article are solely those of the authors and do not necessarily represent those of their affiliated organizations, or those of the publisher, the editors and the reviewers. Any product that may be evaluated in this article, or claim that may be made by its manufacturer, is not guaranteed or endorsed by the publisher.

## Footnotes

^1^ http://abo.com.pl/pl/p/FastGene-apTaq-HotStart-Polymerase/74240
