# Supplementary material for: Salinity matters the most: How environmental factors shape the diversity and structure of cyanobacterial mat communities in high altitude arid ecosystems
Source: Front Microbiol. 2023 Apr 13;14:1108694. doi: 10.3389/fmicb.2023.1108694 (PMC10136773; doi:10.3389/fmicb.2023.1108694)
Supplement: Supplementary file 1 [file Data_Sheet_1.docx]

***Supplementary Material***

**Supplementary Table 1.** Geographical, physical and chemical data of microbial mat samples collected in 2015.

| **Nr** | Subregion | Reservoir | Latitude [DD] | Longitude [DD] | Altitude [m a.s.l.] | Temp [°C] | pH | Salinity [ppt] | C org. [mg/l] | N total [mg/l] | P total [mg/l] | Na [mg/l] | K [mg/l] | Ca [mg/l] | Mg [mg/l] |
| --- | --- | --- | --- | --- | --- | --- | --- | --- | --- | --- | --- | --- | --- | --- | --- |
| **A01** | Chorog | Stream | 37.4928 | 71.5487 | 2380 | 20 | NA | NA | 20 | 1 | 0.003 | 318 | 33 | 132 | 97 |
| **A02** | Bulunkul | Stream | 37.7323 | 72.8913 | 3754 | 26 | 9 | 0.1 | 3 | 1 | 0.02 | 634 | 1 | 7 | 2 |
| **A03** | Bulunkul | Thermokarst pond | 37.7492 | 72.9862 | 3749 | 16 | 7 | 0.2 | 10 | 1 | 0 | 56 | 2 | 20 | 6 |
| **A04** | Bulunkul | Thermokarst pond | 37.6781 | 73.1806 | 3830 | 11 | 8 | 0.2 | 20 | 1 | 0.07 | 176 | 7 | 22 | 5 |
| **A05** | Bulunkul | Thermokarst pond | 37.6785 | 73.1812 | 3830 | 16 | 8 | 0.3 | 21 | 1 | 0.08 | 208 | 8 | 24 | 6 |
| **A06** | Bulunkul | Pool | 37.6782 | 73.1820 | 3834 | 24 | 10 | 11 | 1360 | 13 | 1 | 4500 | 96 | 54 | 7 |
| **A07** | Bulunkul | Thermokarst pond | 37.6780 | 73.1813 | 3837 | 19 | 9 | 1 | 23 | 1 | 0.01 | 158 | 9 | 42 | 36 |
| **A08** | Bulunkul | Stream | 37.6779 | 73.1813 | 3838 | 7 | 7 | 0.2 | 5 | 0.4 | 0.008 | 49 | 2 | 19 | 6 |
| **A09** | Bulunkul | Lake shore | 37.6882 | 73.1879 | 3824 | 18 | 10 | 28 | 4260 | 3 | 2 | 28800 | 1260 | 257 | 19 |
| **A10** | Bulunkul | Lake shore | 37.5520 | 73.1094 | 3966 | 18 | 9 | 45 | 171 | 8 | 0.3 | 18000 | 1340 | 201 | 82 |
| **A11** | Bulunkul | Pool | 37.5520 | 73.1094 | 3966 | 26 | 10 | 22 | NA | NA | NA | 4900 | 386 | 140 | 74 |
| **A12** | Bulunkul | Thermokarst pond | 37.7764 | 72.7387 | 3710 | 8 | 9 | 0.1 | 3 | 0.4 | 0.007 | 4 | 1 | 12 | 3 |
| **A14** | Rangkul | Lake | 38.4397 | 74.1430 | 3797 | 16 | 10 | 2 | 27 | 2 | 0 | 336 | 94 | 44 | 491 |
| **A15** | Rangkul | Stream | 38.4720 | 74.2805 | 3780 | 10 | 8 | 0.3 | 6 | 1 | 0 | 19 | 5 | 59 | 48 |
| **A16** | Rangkul | Pool | 38.4738 | 74.2753 | 3783 | 18 | 8 | 2 | NA | NA | 0.1 | 228 | 31 | 88 | 366 |
| **A17** | Rangkul | Pool | 38.4738 | 74.2753 | 3800 | 11 | 8 | 0.3 | 5 | 1 | 0.003 | 134 | 3 | 36 | 31 |
| **A18** | Rangkul | Pool | 38.4749 | 74.276 | 3817 | 9 | 9 | 0.2 | 4 | 1 | 0.003 | 16 | 5 | 40 | 37 |
| **A19** | Rangkul | Pool | 38.4814 | 74.2792 | 3830 | 16 | 8 | 2 | 93 | 5 | 0.06 | 294 | 37 | 60 | 357 |
| **A20** | Rangkul | Pool | 38.4694 | 74.2795 | 3760 | 11 | 8 | 0.2 | 12 | 1 | 0 | 23 | 4 | 41 | 28 |
| **A21** | Rangkul | Pool | 38.4694 | 74.2795 | 3760 | 15 | 8 | 0.3 | 7 | 1 | 0 | 18 | 4 | 52 | 30 |
| **A22** | Ishkashim | Stream | 36.7092 | 71.5739 | 2600 | 25 | NA | NA | 2 | 0.3 | 0 | NA | NA | NA | NA |

**Supplementary Table 2.** Diversity data for Oxyphotobacteria ASVs in samples collected in 2015 and 2017.

| **Nr** | Percentage among other bacteria | Abundance | Shannon | Pielou | Chao1 |
| --- | --- | --- | --- | --- | --- |
| **A01** | 16 | 1772 | 0.2 | 0.03 | 5 |
| **A02** | 73 | 6295 | 1 | 0.2 | 8 |
| **A03** | 59 | 7095 | 0.3 | 0.1 | 9 |
| **A04** | 63 | 7350 | 1 | 0.2 | 13 |
| **A05** | 49 | 7503 | 2 | 0.2 | 29 |
| **A06** | 39 | 3001 | 0.3 | 0.1 | 4 |
| **A07** | 32 | 2215 | 1 | 0.3 | 11 |
| **A08** | 59 | 4212 | 1 | 0.2 | 5 |
| **A09** | 59 | 7446 | 1 | 0.3 | 7 |
| **A10** | 22 | 13040 | 2 | 0.3 | 17 |
| **A11** | 16 | 929 | 0 | NA | 1 |
| **A12** | 5 | 525 | 0 | NA | 1 |
| **A14** | 16 | 986 | 2 | 0.3 | 16 |
| **A15** | 1 | 305 | 2 | 0.4 | 8 |
| **A16** | 15 | 1485 | 2 | 0.3 | 16 |
| **A17** | 24 | 3700 | 1 | 0.1 | 11 |
| **A18** | 2 | 807 | 2 | 0.4 | 7 |
| **A19** | 1 | 140 | 0.3 | 0.2 | 2 |
| **A20** | 2 | 435 | 1 | 0.3 | 8 |
| **A21** | 35 | 3339 | 3 | 0.3 | 25 |
| **A22** | 1 | 424 | 1 | 0.3 | 6 |
| **E01** | 61 | 9619 | 1 | 0.2 | 15 |
| **E02** | 53 | 4629 | 0.3 | 0.05 | 8 |
| **E03** | 32 | 3171 | 2 | 0.3 | 13 |
| **E04** | 51 | 4596 | 2 | 0.3 | 10 |
| **E05** | 71 | 13118 | 1 | 0.2 | 4 |
| **E06** | 41 | 5323 | 1 | 0.3 | 5 |
| **E07** | 70 | 10126 | 1 | 0.3 | 12 |
| **E08** | 56 | 6725 | 0.4 | 0.3 | 2 |
| **E09** | 20 | 2223 | 0.3 | 0.1 | 5 |
| **E10** | 36 | 4677 | 2 | 0.3 | 17 |
| **E11** | 33 | 3323 | 1 | 0.3 | 10 |
| **E12** | 50 | 3214 | 1 | 0.1 | 14 |
| **E13** | 29 | 1935 | 1 | 0.2 | 5 |
| **E14** | 80 | 4155 | 1 | 0.2 | 10 |
| **E15** | 22 | 1808 | 1 | 0.3 | 5 |
| **E16** | 29 | 59 | 1 | 0 | 2 |
| **E17** | 38 | 1698 | 2 | 0.3 | 16 |
| **E18** | 15 | 1161 | 0.02 | 0.01 | 2 |
| **E19** | 27 | 1643 | 2 | 0.3 | 15 |
| **E20** | 23 | 1399 | 2 | 0.3 | 11 |
| **E21** | 20 | 1826 | 1 | 0.3 | 8 |
| **E22** | 28 | 1559 | 1 | 0.1 | 7 |
| **E23** | 47 | 3725 | 2 | 0.3 | 18 |
| **E24** | 6 | 443 | 2 | 0 | 5 |
| **E25** | 19 | 979 | 1 | 0.2 | 7 |
| **E26** | 23 | 2269 | 2 | 0.3 | 20 |
| **E27** | 37 | 2857 | 2 | 0.2 | 25 |
| **E28** | 16 | 1575 | 2 | 0.3 | 10 |
| **E29** | 3 | 443 | 1 | 0.4 | 3 |
| **E30** | 32 | 3107 | 0.4 | 0.1 | 7 |

**Supplementary Table 3.** 0-1 matrix with Oxyphotobacteria genera identified molecularly (NGS) in each mat type compared with morphological identification (DESS)

| Genus | Amorphous | | Epiphytes  /epiliths | | Jelly-like | | Multilayer hard | | Multilayer soft | | Nostoc | | Non-layered | | Non-layered beneath soil | |
| --- | --- | --- | --- | --- | --- | --- | --- | --- | --- | --- | --- | --- | --- | --- | --- | --- |
|  | DESS | NGS | DESS | NGS | DESS | NGS | DESS | NGS | DESS | NGS | DESS | NGS | DESS | NGS | DESS | NGS |
| Acaryochloris | 0 | 0 | 0 | 0 | 0 | 0 | 0 | 0 | 0 | 0 | 0 | 1 | 0 | 0 | 0 | 0 |
| Aerosakkonema | 0 | 0 | 0 | 0 | 0 | 0 | 0 | 0 | 0 | 0 | 0 | 1 | 0 | 1 | 0 | 0 |
| Alkalinema | 0 | 0 | 0 | 0 | 0 | 0 | 0 | 1 | 0 | 0 | 0 | 1 | 0 | 1 | 0 | 0 |
| Anabaena | 0 | 0 | 0 | 0 | 0 | 1 | 0 | 0 | 0 | 0 | 0 | 0 | 0 | 0 | 0 | 0 |
| Ancylothrix | 0 | 1 | 0 | 0 | 0 | 0 | 0 | 0 | 0 | 0 | 0 | 0 | 0 | 1 | 0 | 0 |
| Aphanocapsa | 0 | 0 | 0 | 1 | 0 | 0 | 1 | 0 | 1 | 0 | 0 | 0 | 1 | 0 | 0 | 0 |
| Arthronema | 0 | 0 | 0 | 0 | 0 | 0 | 0 | 0 | 0 | 1 | 0 | 1 | 0 | 0 | 0 | 0 |
| Calothrix | 1 | 1 | 0 | 1 | 1 | 1 | 1 | 1 | 1 | 1 | 0 | 1 | 1 | 1 | 1 | 1 |
| Chamaesiphon | 0 | 1 | 0 | 1 | 0 | 0 | 0 | 1 | 0 | 1 | 0 | 0 | 1 | 1 | 0 | 1 |
| Chondrocystis | 0 | 0 | 0 | 0 | 0 | 0 | 0 | 0 | 0 | 0 | 0 | 0 | 0 | 0 | 0 | 1 |
| Chroococcidiopsis | 0 | 0 | 0 | 0 | 0 | 0 | 0 | 1 | 0 | 0 | 0 | 1 | 0 | 0 | 0 | 0 |
| Chroococcus | 0 | 0 | 0 | 0 | 1 | 0 | 1 | 1 | 1 | 1 | 0 | 1 | 1 | 1 | 1 | 0 |
| Coleofasciculus | 0 | 1 | 0 | 0 | 0 | 0 | 0 | 0 | 0 | 0 | 0 | 0 | 0 | 0 | 0 | 0 |
| Cyanobacterium | 0 | 1 | 0 | 0 | 0 | 0 | 0 | 0 | 0 | 0 | 0 | 0 | 0 | 1 | 0 | 1 |
| Cyanobium | 0 | 0 | 0 | 0 | 0 | 1 | 0 | 0 | 0 | 1 | 0 | 0 | 0 | 1 | 0 | 1 |
| Cyanothece | 0 | 0 | 0 | 0 | 0 | 0 | 0 | 0 | 0 | 1 | 0 | 0 | 0 | 1 | 0 | 0 |
| Geitlerinema | 0 | 1 | 0 | 1 | 0 | 0 | 0 | 1 | 0 | 1 | 0 | 1 | 1 | 1 | 0 | 1 |
| Gloeobacter | 0 | 0 | 0 | 0 | 0 | 0 | 0 | 1 | 0 | 0 | 0 | 0 | 0 | 1 | 0 | 1 |
| Gloeocapsa | 0 | 0 | 0 | 0 | 0 | 1 | 0 | 0 | 0 | 1 | 0 | 0 | 0 | 1 | 0 | 1 |
| Gloeothece | 0 | 0 | 0 | 0 | 0 | 0 | 0 | 1 | 1 | 1 | 0 | 0 | 0 | 0 | 0 | 0 |
| Gomphosphaeria | 0 | 0 | 0 | 0 | 1 | 0 | 0 | 0 | 1 | 1 | 0 | 0 | 1 | 0 | 0 | 0 |
| Hillbrichtia | 0 | 0 | 0 | 0 | 0 | 0 | 0 | 0 | 0 | 0 | 0 | 0 | 1 | 0 | 0 | 0 |
| Hydrocoleum | 0 | 0 | 0 | 0 | 0 | 0 | 0 | 0 | 0 | 0 | 0 | 0 | 0 | 0 | 0 | 1 |
| Jaaginema | 0 | 1 | 0 | 0 | 0 | 0 | 0 | 0 | 0 | 1 | 0 | 0 | 0 | 0 | 0 | 0 |
| Leptolyngbya | 0 | 1 | 0 | 1 | 1 | 1 | 1 | 1 | 1 | 1 | 0 | 1 | 1 | 1 | 1 | 1 |
| Limnothrix | 0 | 0 | 0 | 0 | 0 | 1 | 0 | 1 | 0 | 1 | 0 | 0 | 0 | 1 | 0 | 1 |
| Lyngbya | 0 | 1 | 0 | 0 | 0 | 0 | 0 | 1 | 1 | 1 | 0 | 0 | 1 | 0 | 1 | 1 |
| Macrochaete | 0 | 0 | 0 | 0 | 0 | 0 | 0 | 1 | 0 | 0 | 0 | 0 | 0 | 0 | 0 | 0 |
| Merismopedia | 0 | 0 | 0 | 0 | 0 | 0 | 1 | 0 | 0 | 0 | 0 | 0 | 0 | 0 | 0 | 0 |
| Microcoleus | 0 | 0 | 0 | 1 | 0 | 0 | 0 | 1 | 0 | 1 | 0 | 1 | 1 | 1 | 0 | 0 |
| Microcystis | 0 | 0 | 0 | 0 | 0 | 0 | 0 | 0 | 1 | 0 | 0 | 0 | 0 | 1 | 0 | 0 |
| Nodularia | 1 | 1 | 0 | 0 | 1 | 1 | 0 | 1 | 0 | 1 | 0 | 1 | 0 | 1 | 0 | 1 |
| Nostoc | 1 | 1 | 0 | 0 | 1 | 0 | 1 | 0 | 1 | 1 | 1 | 1 | 1 | 1 | 1 | 1 |
| Oscillatoria | 0 | 1 | 0 | 1 | 0 | 0 | 0 | 1 | 1 | 1 | 1 | 1 | 1 | 1 | 0 | 1 |
| Phormidium | 1 | 0 | 1 | 0 | 0 | 0 | 1 | 0 | 1 | 0 | 0 | 0 | 1 | 0 | 1 | 0 |
| Planktothrix | 0 | 1 | 0 | 1 | 0 | 0 | 0 | 0 | 0 | 1 | 0 | 1 | 0 | 1 | 0 | 1 |
| Pleurocapsa | 0 | 0 | 1 | 0 | 0 | 0 | 0 | 0 | 0 | 0 | 0 | 0 | 0 | 0 | 0 | 0 |
| Porphyrosiphon | 0 | 0 | 0 | 0 | 0 | 0 | 0 | 0 | 0 | 0 | 0 | 0 | 0 | 1 | 0 | 0 |
| Pseudanabaena | 0 | 0 | 0 | 0 | 0 | 0 | 0 | 0 | 0 | 0 | 0 | 0 | 0 | 1 | 0 | 0 |
| Radiocystis | 0 | 0 | 0 | 0 | 0 | 0 | 0 | 1 | 0 | 1 | 0 | 0 | 0 | 0 | 0 | 0 |
| Rivularia | 0 | 0 | 0 | 0 | 0 | 0 | 0 | 1 | 0 | 1 | 0 | 0 | 0 | 1 | 0 | 1 |
| Spirulina | 1 | 1 | 0 | 0 | 0 | 0 | 0 | 0 | 1 | 1 | 0 | 0 | 0 | 0 | 0 | 0 |
| Synechococcus | 0 | 0 | 0 | 0 | 0 | 0 | 0 | 1 | 0 | 0 | 0 | 0 | 0 | 1 | 0 | 1 |
| Synechocystis | 0 | 0 | 0 | 0 | 0 | 0 | 0 | 0 | 0 | 1 | 0 | 1 | 0 | 1 | 0 | 1 |
| Thermosynechococcus | 0 | 0 | 0 | 0 | 0 | 0 | 0 | 0 | 0 | 0 | 0 | 0 | 0 | 1 | 0 | 0 |
| Tolypothrix | 0 | 0 | 0 | 0 | 0 | 0 | 0 | 1 | 0 | 0 | 0 | 0 | 0 | 1 | 0 | 0 |
| Trichocoleus | 0 | 0 | 0 | 0 | 0 | 0 | 0 | 1 | 0 | 0 | 0 | 0 | 0 | 1 | 0 | 0 |
| Trichormus | 0 | 1 | 0 | 0 | 1 | 1 | 0 | 0 | 0 | 0 | 0 | 0 | 0 | 0 | 0 | 0 |


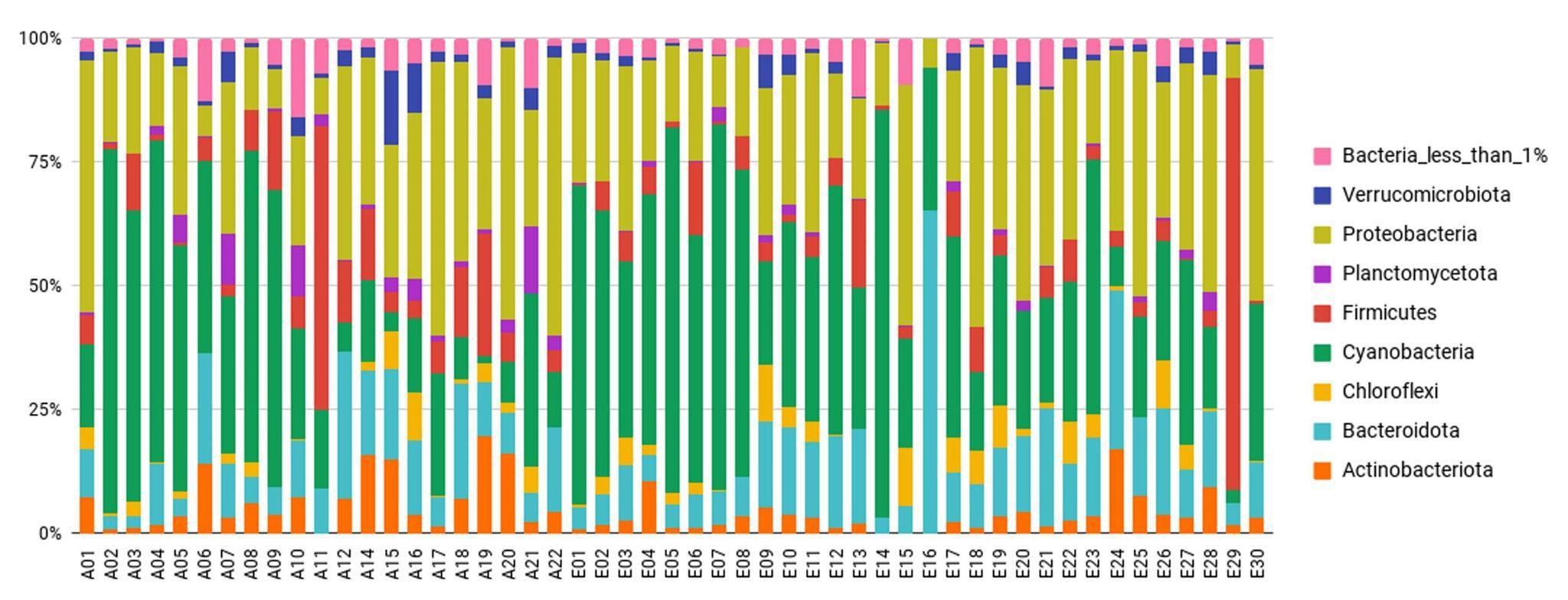


**Supplementary Figure 1.** Bacterial structure of each sample.


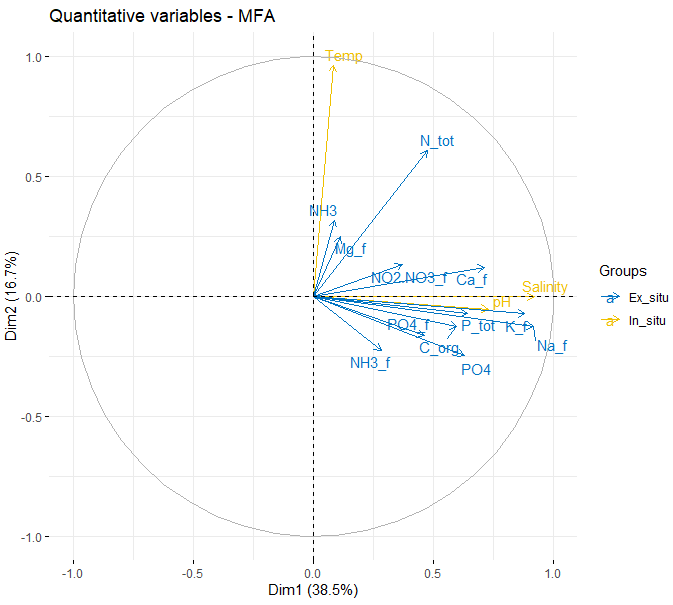


**Supplementary Figure 2.** A variable contribution graph to the MFA in Fig.2. The dataset is divided into two groups: environmental parameters measured *in situ* (temperature, pH, salinity); and nutrient concentration measured *ex situ* (“C_org” - total organic carbon, “N_tot” - total nitrogen, “P_tot” - total phosphorus, “PO4” - PO_4_^3-^, “NH3_f” - NH_3_^+^, “NO2_NO3_f” - NO_2_^-^+NO_3_^-^, “Na_f” - Na^+^, “K_f” - K^+^, “Ca_f” - Ca^+2^, “Mg_f” - Mg^+2^).


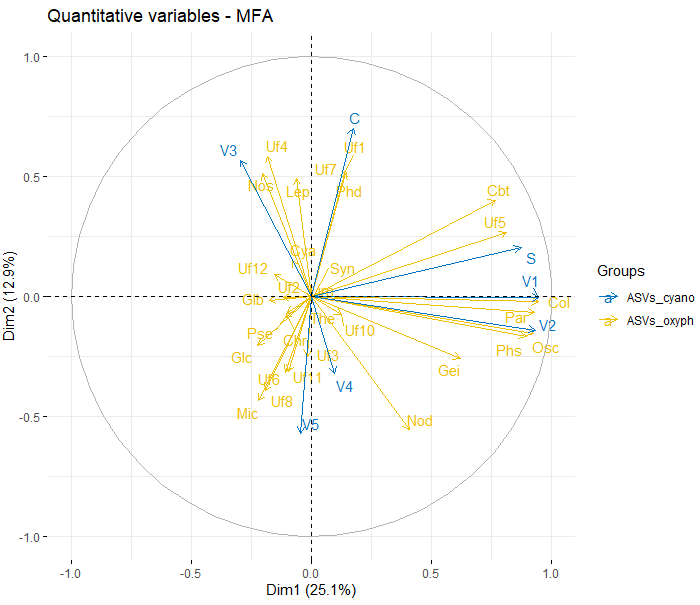


**Supplementary Figure 3.** A variable contribution graph to the MFA in Fig.8. The dataset is divided into two groups: Oxyphotobacterial ASVs (“ASVs_oxyph”) and other ASVs in Cyanobacteria phylum (“ASVs_cyano”). The abbreviations were used as follows: Chr - Chroociccidiopsidaceae; Col - Coleofasciculaceae; Cbt - Cyanobacteriaceae; Cya - Cyanobiaceae; Gei - Geitlerinemaceae; Glb - Gloeobacteraceae; Glc - Gloeocapsaceae; Lep - Leptolyngbyaceae; Lim - Limnotrichaceae; Mic - Microcystaceae; Nod - Nodosilineaceae; Nos - Nostocaceae; Osc - Oscillatoriaceae; Par - Paraspirulinaceae; Phs - Phormidesmiaceae; Phd - Phormidiaceae; Pse - Pseudanabaenaceae; Syn - Synechococcaceae; The - Thermosynechococcaceae; Uf1 - Unknown family (Synechococcales_Incertae_Sedis); Uf2 - Unknown family (Phormidesmiales); Uf3 - Unknown family (Phormidesmiales_uncultured); Uf4 - Unknown family (Oxyphotobacteria_Incertae_Sedis); Uf5 - Unknown family (Cyanobacteriia); Uf6 - Unknown family (Cyanobacteriia_RD017); Uf7 - Unknown family (Cyanobacteriia_SepB-3); Uf8 - Unknown_family_(Cyanobacteriales); Uf9 - Unknown family (Cyanobacteriales_Incertae_Sedis); Uf10 - Unknown family (Cyanobacteriales_Incertae_Sedis); Uf11 - Unknown family (Eurycoccales_Incertae_Sedis); Uf12 - Unknown family (Eurycoccales_uncultured); C - Chloroplast; S - Sericytochromatia; V1 - Vampirivibrionia; V2 - Vampirivibrionia (Gastranaerophilales); V3 - Vampirivibrionia (Obscuribacterales); V4 - Vampirivibrionia (Vampirovibrionales_I); V5 - Vampirivibrionia (Vampirovibrionales_II)
